# Academic achievement and needs of school‐aged children born with selected congenital anomalies: A systematic review and meta‐analysis

**DOI:** 10.1002/bdr2.1961

**Published:** 2021-10-21

**Authors:** Svetlana V. Glinianaia, Ashleigh McLean, Malcolm Moffat, Rebekka Shenfine, Annarita Armaroli, Judith Rankin

**Affiliations:** ^1^ Population Health Sciences Institute, Faculty of Medical Sciences Newcastle University Newcastle upon Tyne United Kingdom; ^2^ Center for Clinical and Epidemiological Research University of Ferrara Ferrara Emilia‐Romagna Italy

**Keywords:** academic performance, birth defects, meta‐analysis, school‐age children, special education

## Abstract

Children with congenital anomalies have poorer intellectual and cognitive development compared to their peers, but evidence for academic achievement using objective measures is lacking. We aimed to summarize and synthesize evidence on academic outcomes and special education needs (SEN) of school‐aged children born with selected major structural congenital anomalies. Electronic databases (MEDLINE, EMBASE, Scopus, PsycINFO, CINAHL, ProQuest Natural Science and Education Collections), reference lists and citations for 1990–2020 were systematically searched. We included original‐research articles on academic achievement in children with non‐syndromic congenital anomalies that involved school test results, standardized tests and/or SEN data. Random‐effects meta‐analyses were performed to estimate pooled mean test scores in mathematics and/or reading where possible and pooled odds ratios (ORs) for SEN in children with severe congenital heart defects (CHDs) and children with orofacial clefts (OFCs). Thirty‐nine eligible studies (*n* = 21,066 children) were synthesized narratively. Sixteen studies were included in meta‐analyses. Children with non‐syndromic congenital anomalies were at a higher risk of academic underachievement than controls across school levels. Children with severe CHD (pooled OR = 2.32, 95% CI: 1.90, 2.82), and children with OFC (OR = 1.38 (95% CI: 1.20, 1.57), OR = 3.07 (95% CI: 2.65, 3.56), and OR = 3.96 (95% CI: 3.31, 4.72) for children with cleft lip, cleft palate and cleft lip/palate, respectively) had significantly higher ORs for SEN than controls. Children with non‐syndromic congenital anomalies underperform academically and have higher SEN rates compared to their peers. Early monitoring and development of differential SEN are important to promote academic progress in these children.

**Abbreviations**
CA: congenital anomaly
CHD: congenital heart defect
CI: confidence interval
CLP: cleft lip and palate
EUROCAT: European Surveillance of Congenital Anomalies
NOS: Newcastle‐Ottawa Quality Assessment Scale
OFC: orofacial cleft
OR: odds ratio
PRISMA: Preferred Reporting Items for Systematic Reviews and Meta‐Analyses
SEN: special education needs, SES, socioeconomic status

## 1 INTRODUCTION

Long‐term (beyond infancy) survival of children born with congenital anomalies (CAs) has improved over the last three decades due to advances in neonatal care and operative interventions (Cassina et al., 2016; Erikssen et al., 2015; Glinianaia et al., 2020; Shin et al., 2012) resulting in an increasing number of children reaching school age. The association with intellectual and learning disabilities has long been established for children with CAs associated with chromosomal and genetic syndromes. Current evidence suggests that children with non‐syndromic/isolated CAs have a higher risk of lower academic achievement and special education needs (SEN) than the reference population. Poorer academic performance is not restricted to children with more severe CAs characterized by lower survival (e.g., severe congenital heart defects, CHDs (Mulkey et al., 2016; Olsen et al., 2011; Oster, Watkins, Hill, Knight, & Meyer, 2017) but is also reported for children with CAs with higher survival (e.g., isolated orofacial clefts, OFCs [Fitzsimons et al., 2018; Fitzsimons et al., 2021; Persson, Becker, & Svensson, 2012; Wehby et al., 2014]) compared to their classmates.

Earlier studies exploring intellectual and cognitive development of children born with CAs used data from parent‐report questionnaires, including a school component, which lacked objective measures of a child's academic performance. Evidence is accumulating from studies using standardized tests measuring academic performance and school tests in larger populations of children with CAs. A summary of the existing evidence of academic performance and SEN in children with different types of CAs, along with the factors associated with educational outcomes, are important for families, health and social care professionals and school team members. This information will help identify timely and effective support during the child's school life to improve academic achievement in this growing population of children and young people.

We performed a systematic review and meta‐analysis of observational studies to summarize and synthesize data on academic outcomes of school‐aged children born with selected major non‐syndromic structural CAs compared to controls or age‐matched referent children. This work was undertaken as part of the European collaborative project EUROlinkCAT (https://www.eurolinkcat.eu/).

## 2 METHODS

### 2.1 Search strategy and selection

The reporting of this systematic review followed the Preferred Reporting Items for Systematic Reviews and Meta‐Analyses (PRISMA) guideline 2009 (Table S1). The review was registered on the PROSPERO database (CRD42017080250). We conducted comprehensive literature searches using: (1) electronic bibliographical databases, (2) reference lists of included papers and relevant literature reviews, (3) citations of included studies (via Google Scholar).

We searched seven electronic databases: MEDLINE, EMBASE, Scopus, PsycINFO, CINAHL, ProQuest Natural Science, and Education Collections. We used keywords and subject headings combining the keywords for the population (child/adolescent/school student/youth), exposure (CAs/birth defects, including specific anomalies, for example, spina bifida, cleft lip), outcome (school/education/academic achievement/performance/special education) and study design (observational studies), incorporating elements of the PICOS (Population/Patient; Intervention/Exposure; Comparator group; Outcome; Study design) framework into our search strategy (Moher et al., 2009) (Table S2). Authors were contacted if any clarification or additional information was needed.

Screening of all titles and abstracts to identify papers for full text review was performed by the first author, while a random 60% sample of records was screened independently by other authors, using the Rayyan software (Ouzzani, Hammady, Fedorowicz, & Elmagarmid, 2016) to ensure consistency in study selection.

### 2.2 Eligibility criteria

Studies were included if they were: (1) observational, peer‐reviewed reporting educational outcomes, that is, academic achievements and/or SEN of school‐aged children (from 4–6 to 18 years old) born with a major structural CA (as defined by the European Surveillance of Congenital Anomalies, EUROCAT [EUROCAT, 2013a], https://eu-rd-platform.jrc.ec.europa.eu/eurocat_en); (2) reporting quantitative measurements of academic performance (e.g., school test scores or questionnaire‐based scores using standardized tests of academic achievement) in children with CAs versus reference/control group or local normative data, or comparing SEN rates with reference groups; (3) published from January 1, 1990 to November 30, 2020 (from 1990 a more inclusive practice towards special education was encouraged [UNESCO, 1994]); and (4) published in the English language.

Studies were excluded if they were: (1) restricted to pre‐school children or adult patients; (2) questionnaire‐based studies exploring quality of life, including school functioning component, executive function, intellectual, cognitive or speech/language development; (3) intervention studies, qualitative studies, case reports or small case series (≤10 cases); (4) reporting educational outcomes in children with conditions other than CAs (e.g., autism, cerebral palsy, intellectual disability) or in children with chromosomal, genetic or teratogenic syndromes known to be associated with lower academic achievement (e.g., Down syndrome, neurofibromatosis, skeletal dysplasia, fetal alcohol syndrome); (5) restricted to a specific patient sub‐group (e.g., preterm births, heart transplant recipients). Based on the results of preliminary searches, we included studies on children with more common and well‐studied CAs; spina bifida (with/without hydrocephalus), CHDs and craniofacial anomalies.

### 2.3 Definitions

We followed the EUROCAT definition for isolated “minor” anomalies defined as “those which do not in themselves have serious medical, functional or cosmetic consequences for the child,” and their definition, diagnosis and reporting vary considerably (EUROCAT, 2013a) to exclude them from eligible CA types for this review.

According to the EUROCAT criteria, we defined severe CHD as including the following CHD types: common arterial truncus, double outlet right ventricle, transposition of great vessels, single ventricle, atrioventricular septal defect, tetralogy of Fallot, pulmonary valve atresia, triscuspid atresia and stenosis, Ebstein anomaly, hypoplastic right heart, aortic valve atresia/stenosis, mitral valve anomalies, hypoplastic left heart, coarctation of aorta, aortic atresia/interrupted aortic arch, total anomalous pulmonary venous return (EUROCAT, 2013b). We consistently use the term “severe CHD” despite variations in the terms used in the included studies. Specific CHD subtypes included in the severe CHD groups in the included studies corresponded to the EUROCAT criteria (see Appendix 1 for studies' definitions).

### 2.4 Data extraction and quality appraisal

Information was extracted on study location, year of publication, study design, study size for cases and comparison groups, and exclusion criteria. We also extracted results of school tests or standardized tests measuring educational outcomes, age of children or school year/grade at assessment and SEN data. The results of the analysis of the association with risk factors were also reported.

The Newcastle‐Ottawa Quality Assessment Scale (NOS) for cohort studies (Wells et al., n.d.) and an amended version for cross‐sectional studies (Modesti et al., 2016), were used to assess study quality. The scale assesses information bias, selection bias and confounding (Table S3). The detailed scores are provided in Table S4.

Full text reviewing, data extraction and quality appraisal of the included studies were performed by two independent reviewers, and identified discrepancies resolved by consensus.

### 2.5 Statistical analysis

Where three or more studies of children with a specific CA reported academic performance in reading and/or mathematics measured by standardized tests, or the percentage with SEN, a random‐effects meta‐analysis was performed to better account for heterogeneity between studies. Further subgroup analysis was performed where possible and heterogeneity was quantified using the *I* ^2^ statistic, with *I* ^2^ > 50% indicating significant ^.^heterogeneity (Higgins, Thompson, Deeks, & Altman, 2003). Publication bias was investigated using Egger's test and funnel plots. Sensitivity analyses, whereby individual studies or subgroups are removed from the analysis and the effect size re‐calculated, were also performed where possible. All analysis was conducted in Stata v16 (StataCorp LLC, 2019).

## 3 RESULTS

### 3.1 Search results

**FIGURE 1:**
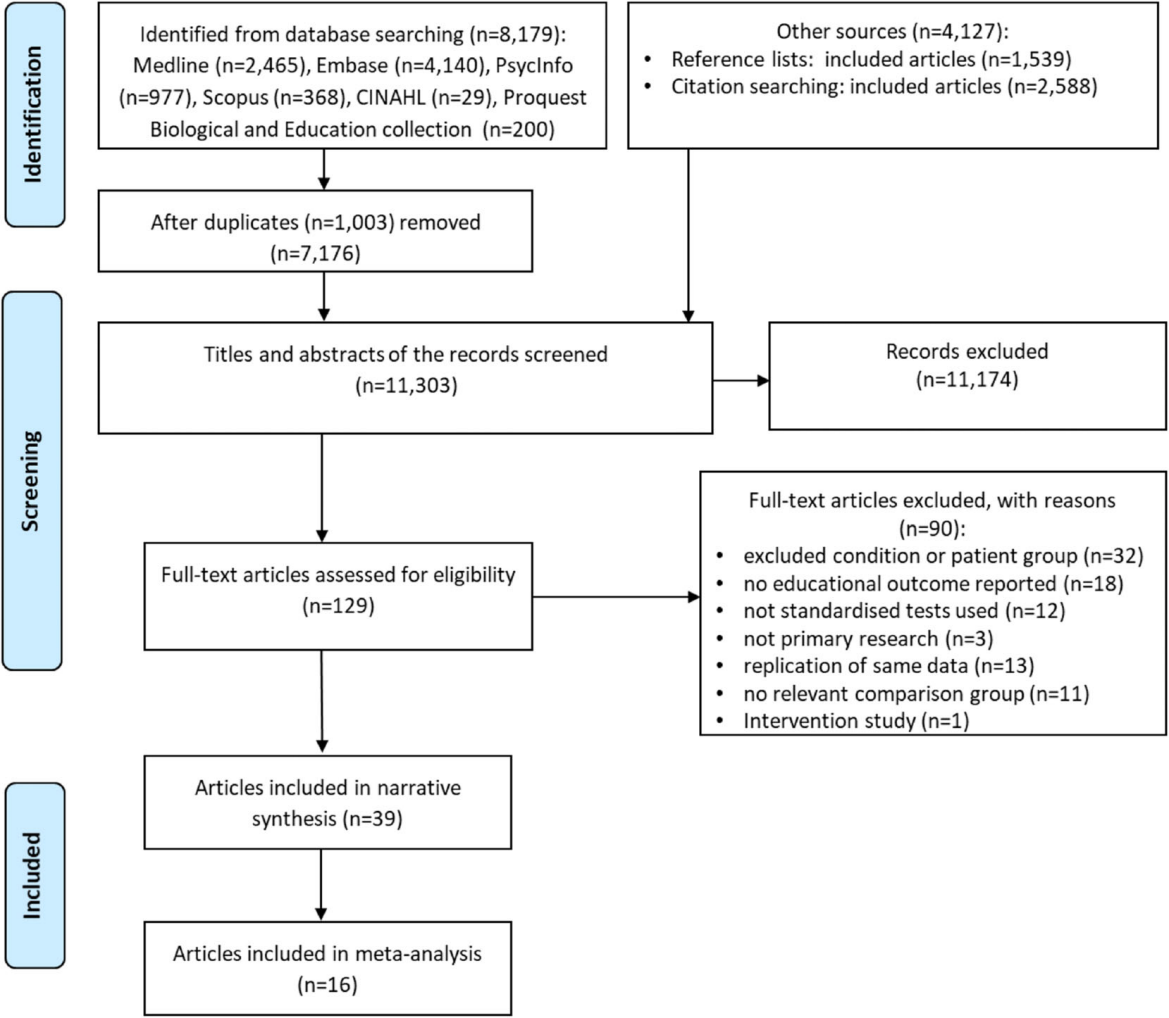
PRISMA flowchart of searches, screening, and study selection

Figure 1 shows the selection of studies from a total of 11,303 records identified for screening titles and abstracts. From 129 studies eligible for full‐text review, 39 met the inclusion criteria, covering a total population of 21,066 children. If publications included an overlapping cohort, the main paper reporting unique educational outcomes of interest (e.g., at different school ages/grades (Fitzsimons et al., 2018; Fitzsimons et al., 2021) or schools tests results in one and SEN data in another paper (Watkins et al., 2018; Watkins et al., 2019)) was included. Sixteen studies were eligible for inclusion in one or more of five meta‐analyses.

### 3.2 Study characteristics

Table 1 shows the description of the 39 included studies and the NOS quality scores. Studies differed by source of CA ascertainment (population‐based registries (*n* = 15), multi‐center (*n* = 8) or single‐center (*n* = 16)), sample size, type of assessment of academic achievement (school tests (*n* = 13), questionnaire‐based using standardized tests (*n* = 19) or SEN data only (*n* = 7)), school age and type of CAs. The included studies reported results for children with the following CAs: spina bifida (with/without hydrocephalus) (*n* = 6), CHDs (*n* = 15) and craniofacial anomalies, including OFCs (*n* = 15), craniosynostosis (*n* = 2), and craniofacial microsomia (*n* = 1) (Table 1). Most studies included isolated or non‐syndromic CAs (*n* = 34), while some also included additional structural or syndromic anomalies, but analyzed them separately (*n* = 4). One study (Fletcher et al., 2005) did not specify whether they excluded children with syndromes; however, as this study excluded children with severe intellectual disability, we included it as well (Table 1). Some studies also excluded specific groups of children known to be associated with lower academic achievement, such as preterm birth (<37 (Collett et al., 2010; Sarrechia et al., 2016), <36 (Hiraiwa et al., 2020) or <34 (Wright & Nolan, 1994) weeks' gestation) or those with low birth weight (<1,500 g (Mulkey et al., 2016) or <2,000 g (Sarrechia et al., 2016; Wright & Nolan, 1994)), analyzed these groups separately (Olsen et al., 2011), or adjusted for these factors in their analyses (Lawley et al., 2019; Oster et al., 2017; Watkins et al., 2019).

**TABLE 1:** Description of included studies listed by congenital anomaly type

Author, publication year, country	Congenital anomaly (CA) type	Study design	Cases, *n*	Type of controls, *n*	Measurement of academic achievement (School test/Questionnaire‐based) or data on special education needs (SEN)	Exclusions	Study quality total score^a^
***Spina bifida***							
Ayr, Yeates, and Enrile (2005), Ohio, USA	Spina bifida (myelomeningocele with hydrocephalus)	Single‐center cross‐sectional	*n* = 24	Controls: children with orthopedic injuries (*n* = 26)	Questionnaire‐based: WRAT‐3, Reading and Arithmetic subtests	Excluded: children with history of severe psychiatric disorder, evidence of abuse or neglect, or any sensory or motor impairment that would preclude administration of the study measures; if their estimated Verbal Comprehension Index or Perceptual Organization Index was ≤80 using WISC‐III.	8
Barf et al. (2004), The Netherlands	Spina bifida with hydrocephalus	Single‐center cross‐sectional	*n* = 118	General population	The percentage of those with SEN in primary (4–11 y) and secondary (≥12 y) schools, change in special education	Excluded two participants with comorbidity that could independently induce physical and/or mental impairments (CHD and chromosome‐13 disorder).	9
Barnes et al. (2006), Ontario, Canada and Texas, USA	Spina bifida myelomeningocele (SBM)	Cross‐sectional data from longitudinal study	*n* = 98	Controls (*n* = 94) had reading and mathematics scores above the 25th percentile	Learning disability group classified using standardized tests: WJTA‐R for word decoding and WRAT‐3 or WJTA‐R for math computations.	Excluded patients with IQ <70.	9
Fletcher et al. (2005), Ontario, Canada and Texas, USA	SBM with hydrocephalus	Cross‐sectional data from longitudinal cohort	*n* = 268	Normative test means (mean = 100 [±15])	Questionnaire‐based: WJTA (WJR Basic Reading, WJR Passage Comprehension, WJR Calculations)	Not reported.	8
Friedrich, Lovejoy, Shaffer, Shurtleff, and Beilke (1991), USA	SBM (with/without hydrocephalus)	Single‐center cross‐sectional	*n* = 68	Normative test means (mean = 100 [±15])	Questionnaire‐based. WRAT	Children outside the age range of 6.5–16 years and children with complicated myelomeningocele (e.g., by CNS infection or bleeding).	7
Wills, Holmbeck, Dillon, and McLone (1990), Chicago, USA	SBM (with/without hydrocephalus)	Prospective cohort	*n* = 89 (*n* = 47 with test results)	Test standardized norms for the age‐matched general population	Questionnaire‐based: WRAT‐R	Excluded patients whose IQ scores on both verbal and performance tests were <70 (*n* = 4) and those who suffered a shunt infection at some time (*n* = 11).	7
***Congenital heart defects (CHD)***							
Bellinger et al. (2015), USA	CHD: Tetralogy of Fallot	Single‐center cross‐sectional	*n* = 91 (out of 184 eligible)	Normally developing referent subjects (*n* = 87)	Questionnaire‐based: WIAT	Excluded patients with conditions that would prevent completion of the study assessments, that is, metal implants, trisomy 21, or lack of reading fluency by the primary caregiver. Those with genetic diagnosis included but analyzed separately.	6
Hiraiwa et al. (2020), Japan	CHD: univentricular	Prospective cohort	*n* = 35 (of 53)	Reference population	Receipt of special education services at 8 years of age	Excluded if: (1) died (*n* = 6); (2) gestational age was <36 weeks (*n* = 2), (3) had a genetic syndrome (*n* = 2), (4) had a history of CNS disease, e.g., brain tumor, brain hemorrhage, and encephalopathy (*n* = 3); (5) had follow up (FU) in another institution (*n* = 5).	8
Lawley et al. (2019), New South Wales, Australia	CHD with a cardiac procedure in the first year of life	Population‐based retrospective cohort	*n* = 396 with linked educational data	Children without surgery in the first year of life (*n* = 261,915)	School test results: NAPLAN programmes in 5 domains: numeracy, grammar, punctuation, writing and spelling (% performing below National Minimum Standard (exempt and Band 1)	Excluded those with severe CAs (except CHD) or cerebral palsy recorded in the first 6 years of life (*n* = 186, 24.2%) and those with ambiguous cardiac diagnosis (*n* = 7).	8
Mahle et al. (2000), Pennsylvania, USA	CHD: hypoplastic left heart syndrome (HLHS)	Cross‐sectional	*n* = 28 (82% of 34) underwent standardized testing, *n* = 115 had SEN data	Age‐matched controls	Questionnaire‐based: WJTA‐R	Patients were eligible if they were born before first January 1992 and underwent staged palliation at the Children's Hospital of Philadelphia for HLHS or its variants. Children with interrupted aortic arch and heterotaxy were excluded.	9
Mlczoch et al. (2009), Austria	Non‐syndromic CHD	Retrospective cohort	School‐aged patients with CHD (*n* = 227) in‐patients in 1996–2005	Austrian background pediatric population	Percentage of children receiving special schooling.	Excluded those with CHD associated with a genetic syndrome.	8
Mulkey et al. (2016), Arkansas, USA	CHD with a cardiac surgery at age < 1 year	Retrospective cohort	School‐aged patients with CHD linked to education database (*n* = 362)	Grade‐matched Arkansas public school students	School test proficiency and the receipt of special education services	Excluded those with birth weight < 1,500 g; patent ductus arteriosus ligation only; born out‐of‐state or not Arkansan; identified genetic condition; neurologic condition.	8
Olsen et al. (2011), Denmark	CHD	Population‐based cohort	*n* = 2,986 (alive at age 13 years)	Random sample of 10 persons per patient matched on sex and year of birth (*n* = 29,246)	Educational attainment (probability of completing compulsory basic school and secondary school)	Sub‐cohort excluding those with extracardiac or chromosomal anomalies and preterm (<37 weeks) analyzed separately.	9
Oster et al. (2017), North Carolina, USA	CHD	Population‐based retrospective cohort	*n* = 2,807 (50%) linked to education records	*n* = 6,355 (59%) controls (random sample matched on birth year)	Reading and maths end‐of‐grade (third grade) examinations; % with SEN	Excluded those where CHD was associated with chromosomal anomalies.	9
Riehle‐Colarusso et al. (2015), Atlanta, USA	Isolated CHD	Population‐based retrospective cohort	*n* = 3,744: critical CHD (*n* = 843), noncritical CHD (2,901)	Referent cohort: children with no CAs (*n* = 860,715)	Receipt of Special Education Services (linked to Special Education Database of Metropolitan Atlanta)	Excluded children with extracardiac CAs, chromosomal abnormalities or genetic syndromes.	9
Sarrechia et al. (2016), Belgium	CHD: univentri‐cular (UVH: HLHS/tricuspid atresia) or biventricular (BiVH)	Cross‐sectional	UVH: *n* = 17, BiVH: *n* = 46	Healthy controls (*n* = 17) matched with UVH cohort (by gender, age and parental education level)	Questionnaire‐based: special education data reported	Excluded from UVH cohort those with perinatal problems, preterm birth (<37 weeks gestational age), birth weight < 2,000 g, other CHDs, genetic and developmental syndromes.	6
Schaefer et al. (2016), Switzerland	Non‐syndromic CHD	Cross‐sectional study	*n* = 204 (of 326 eligible)	General student population of Zurich, (*n* = 38,253)	Questionnaire‐based: the percentage with high, middle and low educational level of the mandatory schooling (7–16 y/o)	Excluded genetic syndromes or a disease known to be associated with learning disabilities, e.g., neurofibromatosis.	7
Simons, Glidden, Sheslow, and Pizarro (2010), Delaware, USA	CHD: Ventricular Septal Defect (VSD)	Cross‐sectional	*n* = 13 (all cases *n* = 31, but *n* = 18 aged < 6 y)	Normative test means (mean = 100 [±15])	Questionnaire‐based: WIAT‐II	Excluded: (1) the presence of chromosomal or genetic syndromes; (2) other medical conditions such as congenital or acquired extracardiac disorders of greater than minor severity, lung disease, spina bifida, omphalocele, or seizure disorder; (3) previous cardiac surgery with bypass; and (4) a history of cyanosis.	8
Wotherspoon et al. (2020), Queensland, Australia	Non‐syndromic CHD after cardiopulmonary bypass surgery	Prospective cohort	*n* = 21	Normative test means (mean = 100 [±15])	Questionnaire‐based: WIAT‐3	Excluded those with syndromes (*n* = 2), lost to FU for various reasons (*n* = 19).	7
Wray & Sensky (2001), UK	CHD: cyanotic and acyanotic	Prospective cohort (assessed before surgery and 12mo after)	CHD (*n* = 47): cyanotic (*n* = 17), acyanotic (*n* = 30)	Healthy children (*n* = 51) similar in mean ages, sex distributions, or parents' SES; standardized test norms	Questionnaire‐based: British Ability Scales (BAS) for reading and arithmetic skills	Excluded from FU assessments: deaths (*n* = 5) and lost to FU (*n* = 7).	9
Wright & Nolan (1994), Victoria, Australia	Cyanotic CHD	Retrospective cohort	*n* = 29 after surgical treatment	Children with cardiac murmurs not requiring treatment (*n* = 36)	Questionnaire‐based: WRAT‐R	Excluded those: (1) with age > 2.5 years at the time of surgery, (2) with non‐cardiac CAs, (3) at greater risk of school difficulties because of recognized complications of their CHD (e.g., postnatal neurological impairment) or for non‐CHD related reasons (e.g., birth asphyxia or seizures, gestational age < 34 weeks or birthweight <2,000 g), (4) neither parent nor child English speaking, (5) non‐resident in Victoria.	7
***Orofacial clefts (OFCs)***							
Bell et al. (2017b), Western Australia	OFCs: cleft palate (CP), cleft lip (CL) and cleft lip and palate (CLP)	Population‐based retrospective cohort	*n* = 532: CL—*n* = 134, CP *n* = 253, CLP—*n* = 145.	Random sample (no OFC, but potentially with other major CAs), matched 4:1 on birth year (*n* = 2,706)	School test results: in WALNA (2,000–3,007) and NAPLAN (2,011 results) programmes to compare the proportion meeting national minimum standards	Excluded: those born outside the testing programs (*n* = 801 with OFC, *n* = 3,462 controls), children with an intellectual disability, classified as exempt or those who died before the Year 3 test (*n* = 126—OFC and *n* = 93 controls). Cases with additional CAs included, but analyzed separately comparing with children with isolated OFCs.	9
Broder, Richman, and Matheson (1998), North Carolina and Iowa, USA	Non‐syndromic OFCs: CP and CLP (CL and CLP)	Retrospective cohort study of cohorts from 2 centers	*n* = 84 for each of two CFC centers (matched by cleft type, age, and sex)	General school population	Standardized national achievement tests (percentage below grade‐level performance defined as < the 25th percentile)	Excluded individuals with mental retardation (*n* = 4) or giftedness (*n* = 2).	8
Chapman (2011), Utah, USA	Isolated OFCs: CLP	Single‐center cross‐sectional	*n* = 28	*n* = 28 age‐matched children without OFC	Questionnaire‐based: TERA‐3	Excluded: those with diagnosed syndrome or other medical conditions, those with sensorineural hearing loss or with cognitive functioning below normal limits.	8
Clausen et al. (2017), Denmark	Isolated OFCs	Population‐based retrospective cohort	OFC: *n* = 558 (5.1% of 588 lost to FU due to deaths and migration)	A 5% sample of the Danish birth cohort with no OFC (*n* = 13,735, 6.4% lost to FU of 14,677).	School results: standardized Danish ninth grade exam	Excluded children with associated anomalies or syndromes.	9
Collett, Leroux, and Speltz (2010), Seattle, USA	Non‐syndromic OFCs	Prospective cohort	CLP: *n* = 29, CP: *n* = 28 (41/57 at age 7 years)	Unaffected controls matched by SES, age and sex (53/77 at age 7 years)	Questionnaire‐based: WJ‐R (early reading subtests)	Excluded children with additional CAs, identified genetic syndromes (e.g., 22q11 deletion), or perinatal problems known to affect cognitive development (e.g., preterm birth) were excluded.	8
Fitzsimons et al. (2018), England	Isolated OFCs	Nationwide register‐based cross‐sectional study	Cases (*n* = 2,802 from 2,924 linked to education database)	General population mean (age‐matched)	School tests: teacher assessed Early Years Foundation Stage Profile (EYFSP) at 5 years of age	Excluded children with non‐isolated OFC, children where cleft type was unknown, those without a linked health or education database record or EYFSP scores.	9
Fitzsimons et al. (2021), England	Isolated OFCs	Nation‐wide register‐based cross‐sectional study	7‐year olds linked to education data (*n* = 3,523)	National figures for the same school level (7‐year olds)	School tests: achieving expected level (≥2) in composite education outcome (5 subjects)—teacher assessment	Excluded children with non‐isolated OFC, children where cleft type was unknown, those without a linked health or education database record.	9
Grewal et al. (2020), UK	Non‐syndromic OFCs	Population‐based Cross‐sectional	Unilateral CLP (*n* = 205)	National average	National tests for educational attainment of 7‐year‐old children in key stage 1 (KS1)	Excluded syndromic cases, those with associated developmental delay affecting cooperation with procedures, refusals from caregivers to participate.	9
Hentges et al. (2011), UK	OFCs: isolated CL with or without CP	Prospective cohort	Total *n* = 93 (44 with neonatal surgery, i.e., 0–28 days, 49 – surgery at 3–4 months of age)	Randomly selected controls (*n* = 77, matched by sex and place of birth)	Questionnaire‐based: Wechsler Quick Test, an achievement test, linked to WIAT, with scores on Basic Reading, Spelling and Math. Reasoning.	Excluded children with other CAs (e.g., syndromes, heart problems) or health problems (e.g., immaturity).	8
Persson et al. (2012), Sweden	Isolated OFCs	Population‐based retrospective cohort	*n* = 1,992, 95.4% of the total eligible individuals (*n* = 2,087)	General student population, *n* = 1,249,404	Final grades of students graduating from compulsory school (16 y/o):	Excluded cases with associated syndromes or other CAs	9
Saervold, Hide, Feragen, and Aukner (2019), Norway	Non‐syndromic OFCs: CP ± CL	Cross‐sectional	*n* = 123	Normative test stanine scores (mean = 5, *SD* = 2)	Word chain test and reading comprehension test: stanine scores (1–9) reported for both	Excluded those with syndromes, developmental difficulties (e.g., developmental delay, autism spectrum disorder), ADHD, specific language impairment, dyslexia.	8
Watkins et al. (2018), N Carolina, USA	Non‐syndromic OFCs	Population‐based retrospective cohort	OFC (*n* = 559)	Random sample of children with no structural birth defects (*n* = 6,822) born during the same period	Linked school records: standardized end‐of‐grade test scores in Reading and Maths from third through eighth grade	Excluded those with syndromes and other major CAs, and death records.	8
Watkins et al. (2019), N Carolina, USA	Non‐syndromic OFCs	Population‐based retrospective cohort	OFC (*n* = 559): CL (*n* = 149), CP (*n* = 194), CLP (*n* = 216)	Random sample of children with no structural birth defects (*n* = 6,822) born during the same period	Linked school records: receiving special education services	Excluded those with syndromes and other major CAs, and death records.	8
Wehby et al. (2014), Iowa, USA	Isolated OFCs	Population‐based retrospective cohort	OFC (*n* = 588): CL (*n* = 219), CP (*n* = 135), CLP (*n* = 234)	Unaffected classmates matched (2:1) by gender, school, grade and month and year of birth (*n* = 1,874)	Iowa standardized school achievement test: ITBS and ITED	Excluded those in kindergarten, grade 1 and grade 12.	9
Yazdy, Autry, Honein, and Frias (2008), Atlanta, USA	OFCs	Population‐based record‐linkage cohort	777 with OFCs; isolated (*n* = 645, 83%)	Children without major CA (*n* = 737,528)	Percentage with SEN	Excluded those with no definite diagnosis of OFC and those with chromosomal anomalies.	9
***Other craniofacial CAs***							
Magge et al. (2002), Connecticut, USA	Non‐syndromic craniosynostosis	Cross‐sectional	*n* = 16 (out of 46 eligible—34.8%)	Normative test means (mean = 100 [±15])	Questionnaire‐based: WRAT‐R	Excluded if aged younger than 6 and older than 16 years, syndromic craniosynostosis, or the presence of additional neurologic complications such as seizures or mental retardation related to hydrocephalus or traumatic brain injury.	6
Speltz et al. (2015), USA	Single‐Suture Cranio‐synostosis (SSC)	Cross‐sectional data from multi‐site longitudinal study	*n* = 182	Children without known SSC matched by age, gender, SES, ethnicity (*n* = 183)	Questionnaire‐based: WRAT‐4; TOWRE; percentage with a learning disability	Prematurity (<34 weeks' gestation), major medical or neurologic conditions (e.g., CHD, seizure disorders, significant health conditions requiring surgery), presence of ≥3 extracranial minor malformations, presence of other major CAs, known syndromes.	9
Speltz et al. (2017), USA & Canada	Craniofacial microsomia	Longitudinal cohort	*n* = 121	Controls (*n* = 315): children without known CAs, not adopted and age‐matched (within 2 months) from the same region	Questionnaire‐based: reading composite scores based on WRAT‐4 (sentence comprehension) and GORT‐4 (Fluency and Comprehension); WJTA‐3 (Writing)	Excluded those with another known syndrome or chromosomal anomaly, cases without microtia and/or at least two craniofacial microsomia‐associated malformations.	9

Abbreviations: AD/HD, attention deficit/hyperactivity disorder; ASD‐II, atrial septum defect secundum type; BAS, British Ability Scales (standardized on a British population); BiVH, biventricular heart defect (ASD‐II/VSD); CFC, Craniofacial Center; CNS, central nervous system; CL, cleft lip only, CLP, cleft lip and palate; CP, cleft palate only; EYFSP, Early Years Foundation Stage Profile; FU, follow up; GORT, Gray Oral Reading Test; HLHS, hypoplastic left heart syndrome; ITBS. Iowa Tests of Basic Skills; ITED, Iowa Tests of Educational Development; KS1, key stage 1; NAPLAN, National Assessment Program ‐ Literacy and Numeracy; OFC, orofacial clefts; SBM, spina bifida myelomeningocele; SEN, special education needs; SES, socioeconomic status; TERA‐3, Test of Early Reading Ability, third edition; TGA, transposition of the great arteries; TOWRE, Test of Word Reading Efficiency; UVH, univentricular heart defect; VSD, ventricular septum defect; WALNA, Western Australian Literacy and Numeracy Assessment; WIAT‐3, Wechsler Individual Achievement Test, third edition; WJTA‐R, Woodcock‐Johnson Tests of Achievement‐Revised; WRAT, Wide Range Achievement Test.

^a^ The Newcastle‐Ottawa Quality Assessment Scale (NOS) for cohort studies (Wells et al.) (maximum 9 scores) and an amended version for cross‐sectional studies (Modesti et al., 2016) (maximum 10 scores) were used to assess the quality of the included studies. Scores of <5 indicated high risk of bias (Luchini, Stubbs, Solmi, & Veronese, 2017).

The NOS scores ranged between 6 and 9 (Table 1); overall, studies had low risk of bias.

### 3.3 Academic achievement and SEN for specific CA types

#### 3.3.1 Spina bifida

**FIGURE 2:**
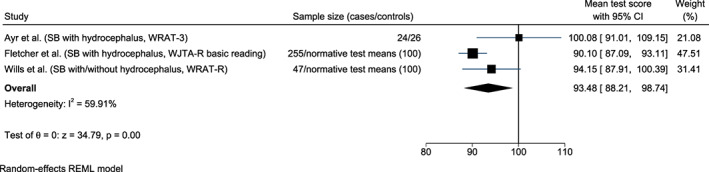
Forest plots showing the mean test scores in reading in children with spina bifida (SB) versus controls. WRAT, Wide Range Achievement Test (norms are standardized to mean = 100, *SD* = 15); WJTA‐R, Woodcock‐Johnson Tests of Achievement‐Revised

**FIGURE 3:**
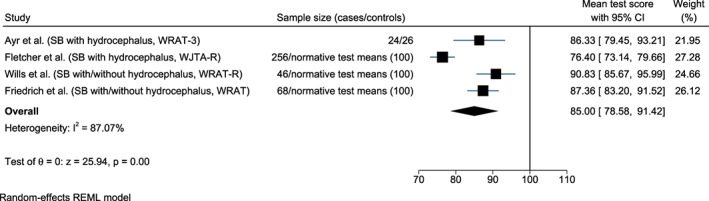
Forest plots showing the mean test scores in mathematics in children with spina bifida (SB) versus controls. WRAT, Wide Range Achievement Test (norms are standardized to mean = 100, *SD* = 15); WRAT‐R, Wide Range Achievement Test‐ Revised; WJTA‐R, Woodcock‐Johnson Tests of Achievement‐Revised

US studies revealed significantly lower scores in children with spina bifida (with hydrocephalus) in mathematics/calculations tests compared to controls or normative standards (Ayr et al., 2005; Fletcher et al., 2005; Friedrich et al., 1991; Wills et al., 1990). However, in reading tests, children with spina bifida performed similarly to controls in two studies (Ayr et al., 2005; Friedrich et al., 1991) (Table 2). Children with spina bifida included in the meta‐analysis had significantly lower mean test scores in reading (93.48, 95% confidence interval (CI) 88.21, 98.74; *I* ^2^ = 59.91%, *n* = 326 (Ayr et al., 2005; Fletcher et al., 2005; Wills et al., 1990)) (Figure 2) and mathematics (85.00, 95% CI 78.58, 91.42; *I* ^2^ = 87.07%, *n* = 394 [Ayr et al., 2005; Fletcher et al., 2005; Friedrich et al., 1991; Wills et al., 1990]) (Figure 3) than controls.

**TABLE 2:** Studies reporting test scores using school tests/exams or standardized tests of academic performance of children born with specific congenital anomalies (CAs) compared to the reference groups

Author, publication year, CA type	School test‐ or questionnaire‐based (QB)	Academic performance test scores, Mean (±*SD*) or Odds ratio (OR) (95% CI)			
		School grade or age^a^, sample (*n*)	Reading^b^	Spelling/writing^b^	Mathematics/numeracy^b^
***Spina bifida***					
Ayr et al. (2005)	QB: WRAT‐3, Reading and	Cases (*n* = 24):11.5 (±2.7)	100.08 (±22.66), *p* > .05 compared to controls	NA	86.33 (±17.20), *p* < .01 compared to controls
	Arithmetic subtests	Controls (*n* = 26): 11.6 (±2.3)	102.38 (±13.19)	NA	102.27 (±11.52)
		Grade 3 or beyond	**WJTA‐R LWI**		**WRAT‐3 Arithmetic/WJTA‐R**:
Barnes et al. (2006)	QB: WJTA‐R and WRAT‐3	RD + MD: mean age 13.1 (±2.7) (*n* = 20) vs. controls (mean 11.8 (±2.2))	Mean (±*SD*): 11 (±7), *p* < .001 lower than every other group	NA	5 (±6) lower than controls [67 (±20)] and NoLD [52 (±22)] (*p* < .001); NS vs. MD group
		MD: 12.0 (±2.7) (*n* = 31)	58 (±22) *p* > .05 vs. 66 (±20) in controls and NoLD	NA	12 (±8) *p* < .001 vs. controls and NoLD
		NoLD: 12.3 (±3.1) (*n* = 47)	73 (±20) vs. 66 (±20) in controls (*p* > .05)	NA	52 (±22) vs. 67 (±20) in controls
Fletcher et al. (2005)	QB: WJTA‐R	Mean = 11.0 (±3.0), range 7–16 year (*n* ranges by test between 253 and 256)	**Basic Reading**: mean (±*SD*): 90.1 (±24.5)		**Calculations**: mean (±*SD*): 76.4 (±26.6)
			**Passage comprehension**: mean (±*SD*): 87.6 (±24.8)		
Friedrich et al. (1991)	QB: WRAT (Reading, Spelling and Arithmetic)	Mean = 9.7 (±2.3) years, range 6.4–15.2 years	102.8 (*n* = 67), NS compared to test norm	97.13 (*n* = 68), NS	87.36 (*n* = 68), (*t* = −5.95, *p* < .05 vs. test norm)
Wills et al. (1990)	QB: WRAT‐R	Range (4–12 years)			
		*n* = 47	94.15 (±21.83), *p* < .05		
		*n* = 46		95.15 (±20.28), NS	
		*n* = 46			90.83 (±17.87), *p* < .001
***Congenital heart defects (CHD)***					
Bellinger et al. (2015)	QB: WIAT	All (*n* = 91): 13–16 year‐olds	Reading composite score: mean (±*SD*): 92.6 (±20.3)	NA	Composite score: mean (±*SD*): 89.5 (±27.6)
Tetralogy of Fallot		Without genetic diagnosis (*n* = 68)	96.1 (±17.8), *p* = .07 vs. population mean		95.1 (±25.6), *p* = .07 vs. population mean
Lawley et al. (2019)—CHD with a cardiac surgery in the first year of life	School tests (NAPLAN)	Grade 3 (7–9 y/o): *n* = 351 for reading, writing, *n* = 353 for numeracy, spelling and grammar	Performing below National Minimum Standard (NMS): 12.2% vs. 5.7% in the reference groups (*p* < .01^c^)	Performing below NMS: 14.2% and 11.6% for spelling and writing respectively vs. 5.4% and 4.2% in the reference groups respectively (*p* < .01^c^)	Performing below NMS: 13.9% vs. 5.4% in the reference group (*p* < .01^c^)
Mahle et al. (2000)—HLHS	QB: WJTA‐R	8.9 ± 2.1 years (*n* = 28 for tests)	median = 85 (range 54–124) vs. mean norm 100 ± 15 (about 1 *SD* below expected values)	NA	Median = 87 (range 47–132) vs. mean norm 100 ± 15 (about 1 *SD* < expected values)
Mulkey et al. (2016)	School tests (Arkansas Augmented Benchmark Examinations)	285 linked to grade 3 and/or 4 achievement tests scores	**Grade 3 literacy** (*n* = 273); proficient: 56% vs. 67%, *p* < .001		**Grade 3 Maths**: (*n* = 272); proficient: 71% vs. 78%, *p* = .006
CHD after cardiac surgery at age < 1 year			**Grade 4 literacy**: (*n* = 238); proficient: 64% vs. 70%, *p* = .046		**Grade 4 Maths**: (*n* = 237); proficient: 65% vs. 73%, *p* = .005
Olsen et al. (2011)	School tests	Compulsory basic schooling: median—16.6 years—CHD (*n* = 2,986), 16.5 years—comparison cohort	**Completed compulsory education**: 85% vs. 87.5% (aHR^d^ 0.79 [95% CI 0.75, 0.82])		
Oster et al. (2017)	School tests	Third grade (9 y/o): *n* = 2,780 for Reading, *n* = 2,798 for Maths	Not meeting standards: CHD −39.9% vs. 31.3% in controls (aOR^e^ 1.38 [1.21, 1.53])	NA	Not meeting standards: CHD −25.5% vs. 21.1% (aOR^e^ 1.14 [1.01, 1.28])
Schaefer et al. (2016)	QB: objective educational outcomes (level of secondary school level I)	7–9 grades of mandatory education (14–16 y/o)	NA	NA	NA
Simons et al. (2010) VSD	QB: WIAT‐2	8.7 ± 2.7 (older cohort: ≥6 y/o): *n* = 13	Word reading: 105.3 ± 10.9, *p* = .104	103.46 ± 14.4, *p* = .346	101.9 ± 6.8, *p* = .687
Wotherspoon et al. (2020)	QB: WIAT‐3	Range (14–17 y), mean = 15 y 4.8 mo (*SD* = 8.4 mo), *n* = 20	Median = 104, *p* = .058 (Wilcoxon signed‐rank test)	102.45 (±18.09), *p* = .55	Maths problem solving (*n* = 21): mean = 95.25 (±14.37); *p* = .079
CHD after CPB surgery		*n* = 21			Numerical operations: median = 81, *p* = .002 (Wilcoxon signed‐rank test)
Wray & Sensky (2001)	QB: BAS—reading and arithmetic tests; Schonell graded spelling test–spelling	**Initial assessment (before surgery)**: All CHD (*n* = 47)	Mean (SEM): 103 (3) vs. 105 (2) in controls (*p* > .05); reading underachievement (score > 1 *SD* below the overall IQ): 25% vs. 18% in controls	**Spelling**: Mean (SEM): 93.3 vs. 98.2 in controls (NS)	Mean (SEM): 107 (3) vs. 105 (2) in controls (NS); arithmetic underachievement (score > 1 *SD* below the overall IQ): 11% vs. 28% in controls
CHD before and after surgery		Cyanotic (*n* = 17)—mean 7.5 (±3.5) years	93 (5), *p* < .05 vs. acyanotic; Reading problem (>1 *SD* below the reading norm): 33% vs. 8% (controls)	81 (6), *p* < .01 vs. acyanotic	98 (5), *p* < .05 vs. acyanotic; arithmetic underachievement: 8% vs. 28% in controls
		Acyanotic (*n* = 30)—7.0 (±3.0) years	107 (4); reading underachievement: 29% vs. 18% (controls)	98 (3)	112 (3); arithmetic underachievement: 13% vs. 28% in controls, NS for controls and acyanotic
		**Follow up assessment**: all CHD (*n* = 35):	102 (4) vs. 107 (3) in controls; reading underachievement: 25% vs. 10% (controls), *p* > .05; Reading problem: 18% vs. 10% (controls)	94 (4) vs. 100 (3) in controls (NS)	106 (3) vs. 103 (3) in controls (NS); arithmetic underachievement: 26% vs. 27% in controls (NS)
		Cyanotic	Reading problem: 50% vs. 10% (controls), *p* = .04, *p* = .02 vs. acyanotic	78 (6), *p* < .01 vs. acyanotic	98 (6), NS vs. acyanotic; arithmetic underachievement: 10% vs. 27% in controls, NS
		Acyanotic	109 (4); NS; reading underachievement: 25% vs. 10% (controls)	101 (4)	109 (3); arithmetic underachievement: 33% vs. 27% in controls, NS
Wright & Nolan (1994) Cyanotic CHD	QB: WRAT‐R—vs. controls who were 0.3 to 0.65 *SD*s below the normative mean	Mean 9.5 (±1.2) y, range (7–11.4 y), (*n* = 29) vs. controls (*n* = 36)	Adjusted mean (*SD*)^f^ 83.9 (17.77) vs. 94.2 (18.50) in controls, *p* = .026	**Spelling**: Adjusted mean (*SD*)^f^: 82.3 (13.17) vs. 90.1 (13.72), *p* = .023	**Arithmetic**: Adjusted mean (*SD*)^f^: 88.6 (12.96) vs. 95.4 (13.49), *p* = .044
***Orofacial clefts (OFCs)***					
Bell et al. (2017b)	School tests—reaching NMS	School year levels 3, 5, 7 and 9 (*n* differs for each level)	OFC vs. no OFC	OFC vs. no OFC	OFC vs. no OFC
		Year level 3 (mean 8.3 years)	WALNA: OR 0.64 (0.36–1.13); NAPLAN: OR 1.32 (0.66, 2.64);	**Spelling**: WALNA: OR 0.83 (0.56–1.23); NAPLAN: OR 0.73 (0.41, 1.31); **Writing**: WALNA (2001–2007): OR 0.89 (0.61, 1.30); NAPLAN: OR 0.81 (0.38, 1.72);	WALNA: OR 0.49 (0.34, 0.72); NAPLAN: OR 0.52 (0.27, 1.01)
		Year level 5 (mean 10.3 years)	WALNA: OR 1.03 (0.56, 1.91); NAPLAN: OR 0.73 (0.44, 1.22);	**Spelling**: WALNA: OR 0.93 (0.61, 1.41); NAPLAN: OR 0.68, (0.40, 1.15); **Writing**: WALNA (2001–2007): OR 0.67 (0.46, 0.98); NAPLAN: OR 0.98 (0.55, 1.73)	WALNA: OR 0.57 (0.38, 0.84); NAPLAN: OR 0.99 (0.50, 1.94);
		Year level 7 (mean 12.2 years)	WALNA: OR 1.31 (0.84, 2.03); NAPLAN: OR 0.36 (0.20, 0.65);	**Spelling**: WALNA: OR 0.89 (0.60–1.33); NAPLAN: OR 0.75 (0.41, 1.38); **Writing**: WALNA (2001–2007): OR 0.68 (0.47, 0.97); NAPLAN: OR 0.41 (0.25, 0.69)	WALNA: OR 1.04 (0.72, 1.51); NAPLAN: OR 0.45 (0.22, 0.94);
		Year level 9 (mean 14.1 years)	WALNA: OR 1.02 (0.41, 2.52); NAPLAN: OR 0.74 (0.39, 1.42);	**Spelling**: NAPLAN only: OR 0.52 (0.31, 0.87); **Writing**: WALNA (2005–2007): OR 2.20 (0.66, 7.34); NAPLAN: OR 0.57 (0.34, 0.96)	WALNA: OR 1.13 (0.43, 3.01); NAPLAN: OR 0.49 (0.24, 0.99);
Broder et al. (1998)	School tests and other education outcomes	Mean 11.4 years (*SD* = 3.2), range 6–18 years; combined 2‐centre data: cleft palate (CP) *n* = 60; cleft lip & palate (CLP): *n* = 108	**Percentage below grade‐level performance**: CP: 50%; CLP: 45%, total: 47%		
CP and CLP		Results for both centers combined	**Prevalence of grade retention**: CP: 27%, CLP: 27%, total: 27%, higher than in the general school population.		
Chapman (2011) CLP	QB: TERA‐3 (reading quotient)	CLP: mean = 5.58 y (±0.58), range 4.92–6.92 (*n* = 28)	99.04 (±12.29) vs. 107.50 (±15.01) (controls), *p* = .025	NA	NA
Clausen et al. (2017)	School tests: ninth‐grade exam scores	Ninth Grade (15–16 y/o)	**Ninth Grade average test score/teacher's score: mean (±*SD*)**		
		Any isolated OFC (*n* = 558, *n* = 455 with available test score)	7.93 (±1.09)/7.98 (±1.09)		
		Cleft lip (CL) (*n* = 164, *n* = 145)	8.10 (±1.04)/ 8.14 (±1.07)		
		CLP (*n* = 211, *n* = 178)	7.83 (±1.12)/7.87 (±1.09)		
		CP (*n* = 183, *n* = 132)	7.85 (±1.10)/7.96 (±1.11)—*p* < .05 vs. controls in adjusted analysis		
		Controls (*n* = 13,735, *n* = 11,921)	7.99 (±1.08)/8.03 (±1.10)		
Collett et al. (2010)		7 years old	Mean (±*SD*)	WJTA‐R Dictation	NA
	QB: WJTA‐R LWI	CLP (*n* = 29)	107 (±17) vs. 100 (±20)	95 (±12) vs. 93 (±16)	
		CP (*n* = 28)	110 (±17) vs. 100 (±20)	102 (±11) vs. 93 (±16)	
	WJTA‐R Passage completion	CLP	107 (±16) vs. 101 (±18)		
		CP	108 (±17) vs. 101 (±18)		
	Reading composite score	CLP	103 (±14) vs. 98 (±17)		
		CP	107 (±14) vs. 98 (±17)		
Fitzsimons et al. (2018)	School tests: EYFSP	5 years old	**Communication, language and literacy**:	**Knowledge and understanding of the world**:	**Mathematical development**:
Isolated OFC	Six areas of learning		Mean *z*‐score: −0.306 (95% CI −0.380, −0.232) vs. general population mean (*z* score = 0)	Mean *z*‐score: −0.24 (95% CI −0.32, −0.16)	Mean *z*‐score: −0.264 (95% CI −0.343, −0.184) vs. general population mean (*z* score = 0)
Fitzsimons et al. (2021)	School tests: achieving expected level (≥2)	7 years old (end of year 2)	**Composite outcome** (for reading, writing, mathematics, science, speaking/listening)		
		CL (*n* = 920)	73.5% (reference)		
		CP (*n* = 1,257)	65.9% (OR 0.70, 95% CI 0.58, 0.84)		
		CLP (*n* = 1,346)	66.1% (OR 0.70, 95% CI 0.58, 0.85)		
Grewal et al. (2020)	School tests:	7 years old	**Reading**	**Writing**	**Mathematics**
Unilateral CLP	achieving expected level (≥2)	Boys (*n* = 135)	82% vs. 88% (national average), *p* = .03	80% vs. 83%, *p* = .4	88% vs. 91%, *p* = .2
		Girls (*n* = 70)	90% vs. 93%, *p* = .3	84% vs. 92%, *p* = .01	94% vs. 94%, *p* = 1.0
	Overall average point score	All (*n* = 205)	**Average point score** (for reading, writing, mathematics and science): 15.38 (±3.42) vs. 16.00 (±3.46) (national average), *p* = .01		
Hentges et al. (2011)	QB: Wechsler Quick Test	Mean = 7.7 (±0.64) years (*n* = 92)	102.99 (±17.52) vs. 104.88 (±14.76) in controls, *p* = .108	**Spelling**: 101.32 (±17.27) vs. 107.07 (±13.80), *p* = .046	103.50 (±15.60) vs. 111.50 (±10.67), *p* = .001
Persson et al. (2012)^g^	School tests	End of compulsory school (16 y/o)	**Swedish (mother tongue)**	**English**	**Mathematics**
	Relative grading system:	CP: *n* = 511	OR 1.26 (0.93, 1.70)	OR 1.41 (1.06, 1.88)	OR 1.40 (1.04, 1.86)
	higher odds of receiving the lowest grade (i.e., 1–2)	CL: *n* = 651	OR 1.13 (0.88, 1.45)	OR 0.94 (0.74, 1.21)	OR 1.13 (0.89, 1.43)
		CLP: *n* = 830	OR 1.11 (0.88, 1.41)	OR 1.11 (0.88, 1.41)	OR 1.06 (0.84, 1.33)
	Reduced odds of getting a pass with	CP	OR 0.99 (0.72, 1.35)	OR 0.85 (0.61, 1.17)	OR 0.67 (0.47, 0.93)
	distinction or excellence	CL	OR 0.72 (0.53, 0.96)	OR 0.83 (0.62, 1.11)	OR 0.93 (0.69, 1.25)
		CLP	OR 0.83 (0.65, 1.06)	OR 0.84 (0.66, 1.06)	OR 0.82 (0.63, 1.06)
	Grade point average		**Grade point average**		
	based on relative	CP	3.06 ± 0.04 vs. 3.24 ± 0.001 in controls (*p* = .002)		
	grading system only	CL	3.12 ± 0.03 vs. 3.24 ± 0.001 *(p* = .003)		
		CLP	3.12 ± 0.03 vs. 3.24 ± 0.001 (*p* = .003)		
Saervold et al. (2019)	QB: Word Chain Test	Fourth or fifth grade, 10‐year‐olds	Mean = 6.3 (±1.61)—upper normal range	NA	NA
CP ± CL	QB: Reading Comprehension		Mean = 5.3 (±1.60)—normal range	NA	NA
Watkins et al. (2018)	School tests	End of third grade performance (not meeting grade‐level standards)	OFC (*n* = 486): 33.1% vs. 31.5% in controls, *p* = .45, aOR^h^ 1.22 (0.99, 1.50)	NA	OFC (*n* = 488): 20.7% vs. 21.1% in controls, *p* = .83, aOR^h^ 1.17 (0.92, 1.48)
Wehby et al. (2014)	School tests	Age range (7–17 y); grades 2–11 (*n* = 588)	55.7 (±28.1) vs. 58.3 (±27.4) in controls, *p* < .01 in regression model		58.0 (±27.9) vs. 61.8 (±27.3), *p* < .01 in regression model
Isolated OFCs			**Composite total** (Reading, Language, Math, Science, Social Studies, and Sources of Information): 58.9 (±27.7) vs. 62.1 (±26.5), *p* < .01		
			**Reading, Language, or Maths < 25th percentile**: 31.4% vs. 25.6%, *p* < .01		
***Other craniofacial CAs***					
*Craniosynostosis*					
Magge et al. (2002)	QB: WRAT‐R	6.4–15.9 years (*n* = 16)	44% (*n* = 7) with RD	38% (*n* = 6) with spelling LD	NA
Speltz et al. (2015)	QB: WRAT‐4	Mean 7.5 years (range 6.9–9.5 years); *n* = 180	**Reading composite**: 105.4 (±16.5) vs. 109.3 (±17.2) (controls), *p* = .03, *p* = .21 in adjusted analysis^i^	**Spelling**: 105.2 (±16.1) vs. 107.2 (±14.3), *p* = .18, *p* = .54 (adjusted^i^)	98.7 (±13.4) vs. 104.1 (±14.6), *p* < .001, *p* = .002 (adjusted^i^)
	TOWRE		**Reading efficiency**: 104.0 (±16.1) vs. 106.6 (±14.4), *p* = .12, *p* = .57 in adjusted analysis^i^		
*Craniofacial microsomia*					
Speltz et al. (2017)	QB: WRAT‐4 (Spelling, Maths); WRAT and GORT (reading composite)	Mean 13 (range 11–17) years (*n* differs by test from 114 to 107)	**Reading composite**: mean 97.2 (±16.7) vs. 103.2 (±14.6), *p* = .001; adjusted^j^ (*p* = .04)	**Spelling**: 104.8 (±16.2) vs. 107.7 (±13.9), *p* = .09, adjusted^j^: *p* = .46	104.7 (±16.1) vs. 108.9 (±15.3), *p* = .02, adjusted^j^ (*p* = .20)
	Writing (WJTA‐3)			**Writing**: 99.4 (±14.2) vs. 104.4 (±12.1), *p* = .001; adjusted^j^ (*p* = .01)	

Abbreviations: aOR, adjusted odds ratio; BAS, British Ability Scales (standardized on a British population); CA, congenital anomaly; CI, confidence interval; CL, cleft lip only; CLP, cleft lip and palate; CP, cleft palate only; CPB, cardiopulmonary bypass; EYFSP, Early Years Foundation Stage Profile; GORT, Gray Oral Reading Test; HLHS, hypoplastic left heart syndrome; LD, learning disability, MD, math disability only (scores below the 25th percentile on the math computation measure but above the 25th percentile on the reading decoding measure); NA, not applicable; NAPLAN, National Assessment Program ‐ Literacy and Numeracy; NMS, National Minimum Standard; NS, not significant (*p* ≥ .05); OR, odds ratio; RD, reading disability only, RD + MD, both reading and math disability (scores below the 25th percentile on both the reading decoding and math measure); TERA‐3, Test of Early Reading Ability, third edition; TOWRE, Test of Word Reading Efficiency; VSD, ventricular septum defect; WALNA, Western Australian Literacy and Numeracy Assessment; WIAT, Wechsler Individual Achievement Test; WJTA‐R, Woodcock‐Johnson Tests of Achievement‐Revised (percentiles); WJTA‐R LWI, Woodcock‐Johnson Tests of Achievement‐Revised Letter‐Word identification (age‐based percentiles); WRAT, Wide Range Achievement Test (norms are standardized to mean = 100, *SD* = 15).

^a^ Mean (±*SD*) or median (IQR) or range.

^b^ The results for the main school subjects (reading, spelling/writing and mathematics) are presented where reported; other subjects are specified in the rows for individual studies if no results for the subjects listed above were reported.

^c^ Provided by authors on request.

^d^ Adjusted for current age, sex, parental income, number of siblings, having a single parent, and parents' highest educational level.

^e^ Adjusted for maternal education, race/ethnicity, public pre‐Kindergarten enrolment, and gestational age.

^f^ Adjusted for group differences in socioeconomic status, gender and maternal education.

^g^ All ORs adjusted for year of birth, maternal age, parity, and maternal education.

^h^ Adjusted for maternal education, race/ethnicity, and public pre‐Kindergarten enrolment.

^i^ Adjusted for age (continuous), gender, socioeconomic status (continuous), maternal IQ (continuous).

^j^ Adjusted for age at assessment (continuous), sex, race/ethnicity (white non‐Hispanic, Hispanic, other), income (categorical), and primary caregiver's highest level of education (categorical).

The need for special education, both in primary and secondary schools, reported in a Dutch study was substantially higher for children with spina bifida associated with hydrocephalus compared to the reference population and those without hydrocephalus (Barf et al., 2004) (Table S5).

Factors that significantly reduced academic achievement (reading and calculations) in 11‐year‐old children with spina bifida (with hydrocephalus) were upper lesion level and Hispanic ethnicity (Fletcher et al., 2005). Shunting for hydrocephalus, IQ ≤85, and wheelchair dependency, were independent predictors of SEN after adjustment for lesion level, annual number of surgical interventions and incontinence (Barf et al., 2004) (Table S6).

#### 3.3.2 Congenital heart defects

Of 15 studies analyzing academic achievement in children with CHDs (*n* = 11,053), 12 included non‐syndromic CHDs, one excluded CHDs associated with chromosomal anomalies (Oster et al., 2017) and two included children with additional anomalies or syndromes but analyzed them separately (Bellinger et al., 2015; Olsen et al., 2011). All but one (Simons et al., 2010) of these studies included severe CHDs. If less severe CHD types were also included, the analysis was stratified by CHD severity (Mlczoch et al., 2009; Olsen et al., 2011; Oster et al., 2017; Riehle‐Colarusso et al., 2015; Schaefer et al., 2016; Wray & Sensky, 2001).

Large studies from the USA, Denmark and Australia using school test results were consistent in reporting poorer academic performance in children with CHD compared to the reference children (Lawley et al., 2019; Mulkey et al., 2016; Olsen et al., 2011; Oster et al., 2017) (Table 2). Third‐grade children (9‐year‐olds) with CHD had poorer performance in reading/literacy and mathematics/numeracy tests compared with their peers (Lawley et al., 2019; Mulkey et al., 2016; Oster et al., 2017). Moreover, when children with severe and non‐severe CHD were analyzed separately, both CHD groups showed significantly poorer academic performance in reading and higher SEN rates (Oster et al., 2017) (Table S6).

Overall, studies using standardized tests were smaller than those using school tests or SEN data. Most of these studies reported poorer scores in reading and mathematics for children with severe CHD compared to controls (Bellinger et al., 2015; Mahle et al., 2000; Wright & Nolan, 1994). However, some reported lower scores in mathematics only (Wotherspoon et al., 2020) or comparable scores in any school subject in children with non‐severe CHD (Simons et al., 2010). In Switzerland, where a structured follow‐up programme of children with CHD is well established, similar percentages of these children completed mandatory school at the high, medium or low educational level compared to controls (Table 2); however, those with severe CHD were over‐represented in the lower level education (*p* = .03) (Schaefer et al., 2016) (Table S6).

**FIGURE 4:**
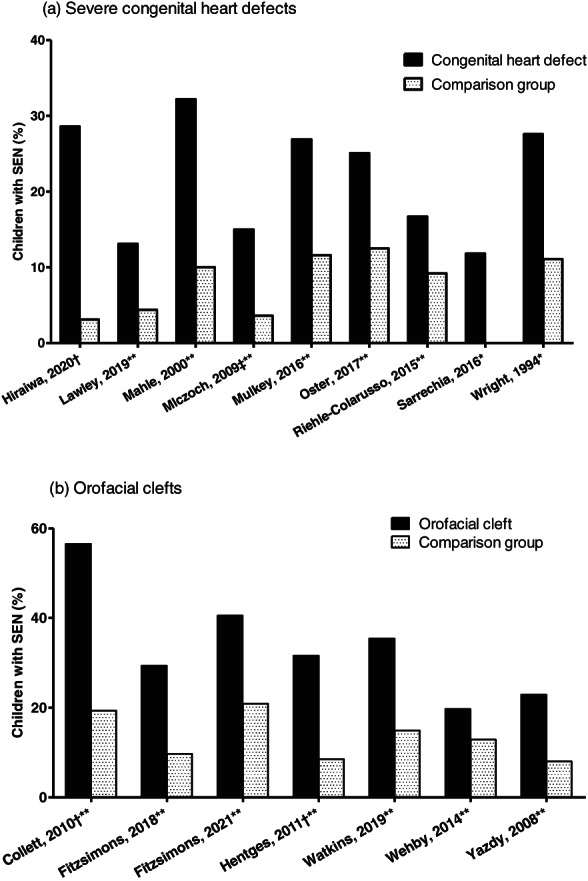
Included studies reporting the percentage of children with special education needs (SEN) in the groups of (a) children with severe congenital heart defects versus a comparison group and (b) children with orofacial clefts versus a comparison group. (a) * *p* > .05, 0% with SEN in controls (*n* = 17); ** *p* < .05; † no *p* value reported; ‡ children with any congenital heart defect are included (86% had cardiac surgery); (b) † the SEN rate for children with cleft lip and palate and for cleft lip with/without cleft palate is shown for Collett et al., 2010 and Hentges et al. (2011), respectively, while the SEN rates for any type of orofacial cleft are shown for other studies; ** *p* < .05

**FIGURE 5:**
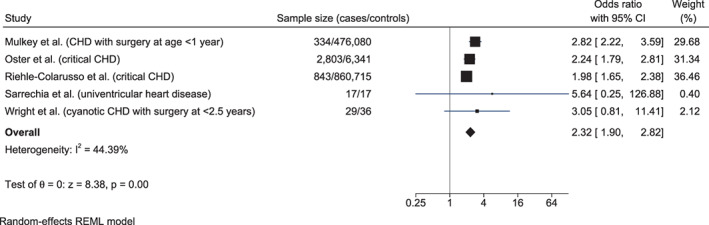
Forest plots showing the odds ratios for special education needs (SEN) for children with severe congenital heart defects (CHD) versus controls

SEN rates in children with CHDs were significantly higher compared to the reference population/controls in all large studies reporting SEN data; in three small studies, the difference was not statistically significant (Sarrechia et al., 2016; Wright & Nolan, 1994) or not reported (Hiraiwa et al., 2020) (Figure 4a). Overall, SEN rates were higher in the USA (Mahle et al., 2000; Mulkey et al., 2016; Oster et al., 2017) compared with Europe (Mlczoch et al., 2009) or Australia (Lawley et al., 2019) for both cases and controls (Table S5). Children with severe CHD from five studies included in the meta‐analysis (*n* = 4,026) (Mulkey et al., 2016; Oster et al., 2017; Riehle‐Colarusso et al., 2015; Sarrechia et al., 2016; Wright & Nolan, 1994) had significantly higher SEN rates than controls (odds ratio, OR 2.32, 95% CI 1.90, 2.82; *I* ^2^ = 44.39%) (Figure 5). This result was robust to the exclusion of individual studies in sensitivity analysis, and there was no evidence of publication bias (Table S7(1)).

Presence of extracardiac CAs (Oster et al., 2017), genetic diagnosis (Bellinger et al., 2015) and CHD severity (Oster et al., 2017; Riehle‐Colarusso et al., 2015; Schaefer et al., 2016; Wray & Sensky, 2001) were major risk factors of lower academic achievement or higher SEN rates (Table 2, Table S6). Other significant predictors of poorer academic achievement included factors related to surgery and hospitalizations: longer duration of hospitalization (Mulkey et al., 2016), >4 re‐hospitalizations in their first six years (Lawley et al., 2019) and ≥2 complications at first surgery (Bellinger et al., 2015), adjusted in all studies for socioeconomic status (SES) or parental education. Receipt of free school meal as a proxy of lower SES (Mulkey et al., 2016), and low level of parental education (Lawley et al., 2019; Mulkey et al., 2016), were also significantly associated with lower academic achievement. Low birth weight was associated with underachievement in writing and numeracy after adjustment for parental education, student language at home and some clinical factors (Lawley et al., 2019) (Table S6).

#### 3.3.3 Craniofacial anomalies—orofacial clefts

In 15 studies of children with OFCs, nine used school tests or teacher's assessment at different age groups, four used standardized tests (Chapman, 2011; Collett et al., 2010; Hentges et al., 2011; Saervold et al., 2019), two analyzed SEN data only (Watkins et al., 2019; Yazdy et al., 2008), with five reporting both academic and SEN results (Collett et al., 2010; Fitzsimons et al., 2018; Fitzsimons et al., 2021; Hentges et al., 2011; Wehby et al., 2014) (Table 2, Table S5). Studies in Europe, USA and Australia using school test results were consistent in reporting lower achievement in children with OFC in various school age groups compared to controls (Bell et al., 2017b; Clausen et al., 2017; Fitzsimons et al., 2018; Fitzsimons et al., 2021; Grewal et al., 2020; Persson et al., 2012; Watkins et al., 2018; Wehby et al., 2014), however, the significant differences were not consistent for all academic domains and OFC types (Table 2). Findings from most studies of children with isolated OFC agreed that poorer academic outcomes were associated with cleft type, reporting a lower risk for children with cleft lip only, who often performed similarly to controls (Bell et al., 2017b; Clausen et al., 2017; Fitzsimons et al., 2018; Fitzsimons et al., 2021; Persson et al., 2012; Watkins et al., 2018; Wehby et al., 2014). There was less consistency for cleft palate and cleft lip with palate (CLP), with significantly higher risks for both groups reported in some studies (Fitzsimons et al., 2018; Fitzsimons et al., 2021), but for cleft palate only in others (Persson et al., 2012; Wehby et al., 2014) or CLP (Watkins et al., 2018). Longitudinal studies showed that children with isolated OFC were at higher risk of lower performance across all academic areas and grade levels, from elementary to high school (7–17 years), in particular for children with cleft palate (Wehby et al., 2014) (Table S6). In a recent Danish study, the OFC type was concluded to be more important for academic performance than timing and number of exposures to surgery and anesthesia (Clausen et al., 2017).

Recent population‐based English studies reported that academic achievement in 5‐year‐old (Fitzsimons et al., 2018) and 7‐year‐old (Fitzsimons et al., 2021) children with isolated OFC, particularly in those with palate involvement, was significantly lower in all school subject areas compared to the national average (Table 2). They also reported a significantly higher SEN rate in both age groups, with higher rates for children with a cleft involving the palate (Fitzsimons et al., 2018; Fitzsimons et al., 2021) (Table S5, Figure 4b). Both cleft type and school absence were independent risk factors for lower academic achievement after adjustment for SES and child's sex (Fitzsimons et al., 2021). SEN rates in children with OFC were also significantly higher compared to controls in US studies (Collett et al., 2010; Hentges et al., 2011; Watkins et al., 2019; Wehby et al., 2014; Yazdy et al., 2008) (Figure 4b), ranging between 19.7% (Wehby et al., 2014) and 35.4% (Watkins et al., 2019) for all OFCs, rising to 56.5% for CLP and 41% in cleft palate (Collett et al., 2010). The higher SEN rates were consistent for children with non‐syndromic OFC across all school levels (Wehby et al., 2014), remaining significant after exclusion of those receiving speech and language services (Watkins et al., 2019).

**FIGURE 6:**
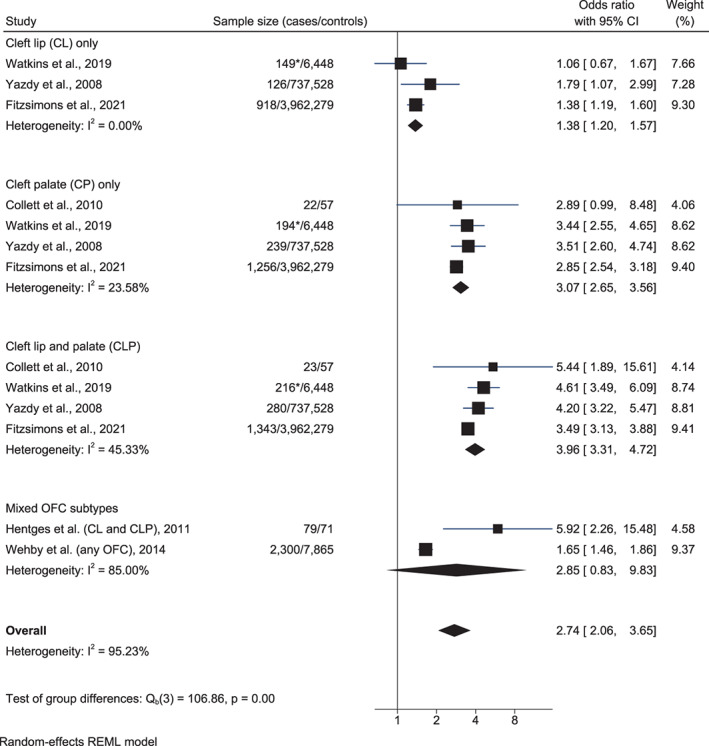
Forest plots showing the odds ratios for special education needs (SEN) for children with isolated/non‐syndromic orofacial clefts (OFCs), by OFC type (cleft lip (CL), cleft palate (CP), cleft lip and palate (CLP) and mixed) versus controls. *The overall number of children by OFC type is given, as the number with known SEN status, which is lower by a total of 36 cases, is not reported and could not be obtained from the authors

Children with OFCs from six studies included in the meta‐analysis (*n* = 7,145) (Collett et al., 2010; Fitzsimons et al., 2021; Hentges et al., 2011; Watkins et al., 2018; Wehby et al., 2014; Yazdy et al., 2008) had significantly higher SEN rates than controls (OR 2.74, 95% CI 2.06, 3.65) (Figure 6). Odds of SEN were significantly higher for all OFCs types; cleft lip: 1.38 (95% CI 1.20, 1.57; *I* ^2^ = 0.00%), cleft palate: OR 3.07 (95% CI 2.65, 3.56; *I* ^2^ = 23.58%), CLP: OR 3.96 (95% CI 3.31, 4.72; *I* ^2^ = 45.33%) with relatively low heterogeneity between studies. The high heterogeneity of the overall effect size estimate (*I* ^2^ = 95.23%) confirms the importance of distinguishing the risk between different OFC types. These results were robust to the exclusion of individual studies and subgroups in sensitivity analysis, and there was no evidence of publication bias (Table S7(2)).

**FIGURE 7:**
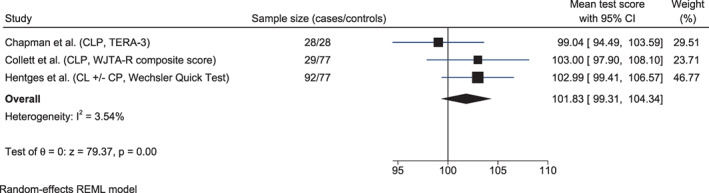
Forest plots showing the mean test scores in reading in children with non‐syndromic orofacial clefts (cleft lip and palate (CLP) or cleft lip (CL) ± cleft palate (CP)) versus controls. TERA‐3, Test of Early Reading Ability, third edition; WJTA‐R, Woodcock‐Johnson Tests of Achievement‐Revised

Studies using standardized tests of academic performance in 7‐year‐old children with cleft lip with/without cleft palate reported significantly lower scores in spelling and mathematical reasoning compared to controls, but not in reading (Hentges et al., 2011) (Table 2). The meta‐analysis of three studies (*n* = 149) (Chapman, 2011; Collett et al., 2010; Hentges et al., 2011) revealed a higher mean reading test score in children with CLP or cleft lip with/without cleft palate than in controls (101.83, 95% CI 99.31, 104.34; *I* ^2^ = 3.54%), but the difference was not statistically significant (Figure 7).

In addition to OFC type and school absence, other factors negatively affecting school attainment in children with OFC were: presence of associated anomalies (Bell et al., 2017b), male sex (Broder et al., 1998; Clausen et al., 2017), late timing of cleft repair (at 3–4 months versus neonatal) (Hentges et al., 2011), delayed speech (Chapman, 2011) and lower SES (Fitzsimons et al., 2018). Reduced speech intelligibility, poor oral health and ≥3 functional problems were associated with lower average point score in 7‐year old children with unilateral CLP after adjustment for birth month, child's sex and SES (Grewal et al., 2020). SES measures such as area‐based deprivation index (Bell et al., 2017b; Fitzsimons et al., 2018; Fitzsimons et al., 2021; Grewal et al., 2020), free school meal (Fitzsimons et al., 2018; Fitzsimons et al., 2021), family social class (Collett et al., 2010) or parental education (Clausen et al., 2017; Watkins et al., 2018; Wehby et al., 2014) were included in all multivariate analyses of the association between different OFC types and education outcomes (Table S6). Differences in SEN rates by ethnicity reported by two US studies were inconsistent (Watkins et al., 2019; Yazdy et al., 2008).

#### 3.3.4 Other craniofacial anomalies

A multi‐center US study of children with single‐suture craniosynostosis reported significantly lower scores in reading composite test and mathematics (Speltz et al., 2015). Unicoronal type of craniosynostosis was a risk factor for significantly lower scores in reading, spelling and mathematics compared to sagittal type after adjustment for age, sex, SES and maternal IQ (Speltz et al., 2015) (Table S6).

Children with craniofacial microsomia scored lower than test norms in reading, writing and mathematics; differences remained significant for reading and writing after adjustment for confounders (Speltz et al., 2017) (Table 2).

## 4 DISCUSSION

Most included studies of children with spina bifida with hydrocephalus, CHDs and OFCs found some degree of academic underperformance or higher SEN rates in these children compared to the referent children regardless of settings and measures of academic outcomes. The meta‐analysis results showed significantly higher pooled ORs for SEN in children with severe CHDs and children with OFCs, in particular for those involving palate, than in controls, with relatively low heterogeneity between studies. Lower academic achievement was reported across a number of academic domains, showing consistency across the school grades and levels in longitudinal studies and in studies analyzing different school ages. Longer school absence, specific anomaly type resulting in higher severity, presence of associated anomalies, some surgery‐related factors, socioeconomic deprivation and lower parental education were the leading factors negatively affecting academic outcomes.

Neurodevelopmental and cognitive impairment found in toddlers and pre‐school children with spina bifida and/or hydrocephalus that manifest in poor school achievement originates from fetal abnormal brain development typical for these CAs (Dennis & Barnes, 2010; Juranek & Salman, 2010). Indicators of brain injury resulting in poorer neurodevelopmental and educational outcomes are also described for such CAs requiring corrective surgeries in infancy such as severe CHDs (Gaynor, 2014; Griffin, Elkin, & Smith, 2003; Wray, 2006) and OFCs (Gallagher & Collett, 2019). Earlier studies of children with CHDs linked poorer neurodevelopmental outcomes with anesthesia‐related neurotoxicity and other surgery‐related factors, including cardiopulmonary bypass, therefore focusing on intra‐ and perioperative neuroprotection and neuromonitoring of infants with severe CHDs to prevent brain injury in these children (Hirsch et al., 2012). However, there is accumulating evidence that factors preceding surgery, that is, delayed intrauterine brain maturation and white matter injury resulting from impaired fetal hemodynamics due to CHD, consequent brain immaturity at birth and longer time before surgery, are primary major risk factors underlying hypoxic brain injury and subsequent poor neurodevelopmental outcomes after surgery (Bolduc, Lambert, Ganeshamoorthy, & Brossard‐Racine, 2018; Gaynor, 2014). Therefore, preventive strategies are suggested to be more effective if initiated antenatally (Gaynor, 2014). Large recent population‐based studies reported that while CHD severity was a major predictor of lower educational achievement (Oster et al., 2017; Schaefer et al., 2016; Wray & Sensky, 2001), non‐severe CHDs were also associated with poorer academic achievement in two different age groups of children (Olsen et al., 2011; Oster et al., 2017). This suggests that children with mild/moderate CHDs also need assessment, monitoring and support from early childhood to adolescence.

Recent studies are strengthened by use of population‐based data and longitudinal design allowing follow up of school attainment of children with CAs compared to earlier smaller cross‐sectional studies. In a longitudinal study, children with OFC, particularly with cleft palate, showed consistently lower achievement across school ages for reading, language and mathematics (Wehby et al., 2014) with persistent, low achievement trajectories after adjusting for SES (Wehby, Collett, Barron, Romitti, & Ansley, 2015), in contrast to earlier reports of lower rates of learning disability in adolescents compared to younger children (Richman, McCoy, Conrad, & Nopoulos, 2012). Higher odds of receiving lower graduation grades and not receiving leaving certificates by 16‐year‐old Swedish adolescents with OFCs compared to the general population (Persson et al., 2012) confirm persistent low achievement and the need for early screening and academic support in these children. Thus, receipt of SEN services decreased the likelihood of third‐grade retention (9‐year‐olds) of children with OFC in an US study (Watkins et al., 2019).

There was strong evidence across the included studies that children with OFC involving palate are at a consistently higher risk of lower education outcomes than children with cleft lip only, irrespectively of school age or measures used (Bell et al., 2017b; Broder et al., 1998; Chapman, 2011; Clausen et al., 2017; Collett et al., 2010; Fitzsimons et al., 2018; Fitzsimons et al., 2021; Persson et al., 2012; Watkins et al., 2018; Wehby et al., 2014). Both cleft type and school absence were independent predictors for underachievement in children with OFC (Fitzsimons et al., 2021). Neither longer school absence, nor socioeconomic differences could explain poorer school attainment in an English study of 7‐year old children with cleft palate and CLP compared with children with cleft lip (Fitzsimons et al., 2021). An Australian study found that school absence adversely affected academic performance in all secondary school children, not being differentially worse for children with OFCs (Bell et al., 2017a). Surgery‐related factors, such as exposure to anesthesia and larger number of operations for OFC correction, had little impact on poorer academic performance compared to OFC type (Clausen et al., 2017). This was recently confirmed in large samples of healthy children that neither early (<2 years) exposure to anesthesia nor multiple exposures were major risk factors for adverse neurodevelopmental outcomes (Graham, 2017).

Much higher SEN rates, especially for children with OFC involving palate, were also consistently reported (Collett et al., 2010; Fitzsimons et al., 2018; Fitzsimons et al., 2021; Watkins et al., 2018; Wehby et al., 2014) and confirmed by the results of our meta‐analysis. Although the most common was speech, language and communication services associated with developing reading skills (Collett et al., 2010; Fitzsimons et al., 2018; Watkins et al., 2019), after exclusion of children with this SEN type, the SEN rate in third‐grade children (9‐year‐olds) with non‐syndromic OFC was still higher than in controls (Watkins et al., 2019). More research is needed to explore the etiology of educational underachievement in children with non‐syndromic OFCs, as a recent genome‐wide association study meta‐analysis of non‐syndromic clef lip/palate found little evidence for shared genetic etiology or causal relationship between this OFC type and educational attainment (Dardani et al., 2020).

For children with either CHD or OFC, CA severity was a major risk factor of lower academic achievement and higher SEN rates consistently reported in the included studies after adjustment for SES. The adjustment for SES is a valid approach recommended for any research on school performance due to a well‐established SES‐achievement relationship and the importance of social and economic context in understanding school achievement (Sirin, 2005).

Major strengths of our systematic review include a rigorous search strategy and comprehensive literature searches using multiple sources. Our search strategy incorporated elements of the PICOS framework based on strict inclusion criteria, was piloted using Medline, then refined and retested to ensure appropriate inclusiveness. We also manually searched the reference lists and citations of included papers, thus increasing the identification of relevant papers (Papaioannou, Sutton, Carroll, Booth, & Wong, 2010). Screening of titles and abstracts of identified records was performed by several authors and the results were compared to enable consistency in study inclusion. Detailed reviewing of the full texts of potentially suitable papers and data extraction were performed independently and in duplicate. We used an established quality assessment tool as part of the critical appraisal process, which was amended for cross‐sectional studies, following advice from an information scientist.

We started literature searches from 1990 to ensure relative consistency among included studies towards special education, as in the mid‐1990s more inclusive education for children with intellectual disability and SEN was encouraged following the 1990 World Conference on Education for All and the 1994 UNESCO statement (UNESCO, 1994). We limited the included studies to those with isolated/non‐syndromic anomalies to avoid bias, as multiple anomalies, chromosomal and genetic syndromes are known to be associated with poorer academic achievement. Our inclusion criteria restricted measurements of academic attainment to the school tests or standardized tests of education achievement, as questionnaire‐based subjective measures do not always coincide with the objective measures, because parents of children with a major CA (e.g., severe CHD) tend to overestimate their child's school performance due to their lower expectations (Mahle et al., 2000).

However, there were several limitations. Due to substantial study heterogeneity in measures of academic achievement for a specific academic domain or school age, we had to restrict the meta‐analysis to studies on relatively common CAs (e.g., OFCs) with sufficient data for a specific subgroup (e.g., CLP) that used age‐standardized tests with unified test norms (mean and *SD*) in a specific academic domain (e.g., reading). Due to the small number of studies suitable for meta‐analysis, subgroup analysis was rarely feasible, and exploration of possible publication bias with funnel plots and Egger's test is generally discouraged in meta‐analyses that include fewer than ten studies. Similarly, the small number of included studies precluded the use of meta‐regression to explore the high heterogeneity seen in some analyses. The included studies did not always give substantial detail of their definition and selection criteria of isolated and non‐syndromic CAs, therefore we cannot exclude a proportion of children with associated CAs in some included studies which may have affected the results. In addition, we were unable to assess the contribution of such potential pathways of academic underachievement in children with specific CAs as surgery and anesthesia or potential mediation effects via some psychological factors (self‐confidence or self‐efficacy in school) or school absence for medical reasons due to lack of this information in the included studies.

## 5 CONCLUSION

This systematic review reports that children with selected non‐syndromic CAs are at a higher risk of academic underperformance across several fundamental school subjects, which may remain persistent over the school levels, and their need for special education services is significantly higher than in reference populations. Evidence from population‐based studies of non‐syndromic CAs confirms that lower academic achievement in children with CAs is not limited to those with chromosomal or genetic syndromes. As the risks significantly differ for specific CA types, early screening, identification and development of differential SEN are important to support children and families to promote their academic proficiency across all school levels. Further population‐based studies should aim to involve high‐quality register‐based data of isolated CAs, including more rare CA types, and linking with objective longitudinal pre‐school and academic outcome data. This would help further understanding of the origin of their academic underperformance, the association with pre‐school development, the identification of specific groups of children at risk and the introduction of timely and targeted interventions to inform education and social services and plan appropriate resources.

## CONFLICT OF INTEREST

The authors declare no conflict of interest.

## Supporting information


**TABLE S1** PRISMA checklist.

**TABLE S2** Search terms and search results in electronic databases Medline and Embase.

**TABLE S3** Adapted Newcastle‐Ottawa Quality Assessment Scale for cohort and cross‐sectional studies.

**TABLE S4** Quality assessment scores of the included studies using the adapted versions of the Newcastle‐Ottawa scale for cohort and cross‐sectional studies.

**TABLE S5** Studies reporting data on special education needs (SEN) or type of school for children born with specific congenital anomalies compared to the reference groups.

**TABLE S6** Risk factors associated with poorer academic performance and/or special education needs (SEN) in children with specific congenital anomalies (CAs).

**TABLE S7** Sensitivity analyses and tests for publication bias for papers included in the meta‐analysis of special education needs (SEN) in children with (1) congenital heart defects (CHD) and (2) orofacial clefts (OFC).

## Appendix A. Definitions of severe congenital heart defects (CHD) in the included studies

Mulkey et al. (2016): CHD type requiring surgery at age <1 year was divided into four categories: left‐ventricular outflow tract obstruction (LVOTO; including interrupted aortic arch type A, aortic stenosis, coarctation, and hypoplastic left heart syndrome), right‐ventricular outflow tract obstruction (RVOTO; including pulmonary atresia, Ebstein's anomaly, tricuspid atresia, and pulmonary valve stenosis), conotruncal defects (including interrupted aortic arch type B, truncus arteriosus, dextro‐transposition of the great arteries, tetralogy of Fallot, and double‐outlet right ventricle), and other (i.e., anomalous pulmonary venous return, atrioventricular septal defects, ventricular and atrial septal defects, heterotaxy, and complex [typically three or more major phenotypes]).

Olsen et al. (2011): severe CHD: common arterial trunk, transposition of great vessels, tetralogy of Fallot, atrioventricular septal defect, anomalies of heart valve, other malformations of great arteries, and malformations of great veins; minor‐to‐moderate severity CHD: ventricular septal defect, atrial septal defect, patent ductus arteriosus, and coarctation of aorta.

Oster et al. (2017): critical CHD: coarctation of the aorta, d‐transposition of the great arteries, double outlet right ventricle, Ebstein anomaly, hypoplastic left heart syndrome, interrupted aortic arch, pulmonary atresia, single ventricle, tetralogy of Fallot, total anomalous pulmonary venous return, tricuspid atresia, or truncus arteriosus.

Riehle‐Colarusso et al. (2015): critical CHD: coarctation of the aorta, d‐transposition of the great arteries; double‐outlet right ventricle, Ebstein anomaly, hypoplastic left heart syndrome, interrupted aortic arch, pulmonary atresia, single ventricle, tetralogy of Fallot, total anomalous pulmonary venous return, tricuspid atresia, truncus arteriosus.

Schaefer et al. (2016): categorized CHD by the Task Force classification system of the American College of Cardiology (Warnes et al., 2001) into simple, moderate and severe. Severe CHD included in the study: criss‐cross heart, double‐inlet left ventricle, pulmonary atresia with/without ventricular septal defect, d‐transposition of the great arteries, l‐transposition of the great arteries, tricuspid atresia, severe hypertrophic cardiomyopathy and other.

Wotherspoon et al. (2020): the following CHD types after cardiopulmonary bypass surgery before 6 months of age: transposition of the great arteries, tetralogy of Fallot, total anomalous pulmonary venous drainage, complex functionally single ventricle, congenitally corrected transposition of the great arteries, ventricular septal defect, valvar pulmonary stenosis.
