# Supplementary material for: Academic achievement and needs of school‐aged children born with selected congenital anomalies: A systematic review and meta‐analysis
Source: Birth Defects Res. 2021 Oct 21;113(20):1431–62. doi: 10.1002/bdr2.1961 (PMC9298217; doi:10.1002/bdr2.1961)

TABLE S2 Search terms and search results in electronic databases Medline and Embase.

Medline (search on 19/11/2019)


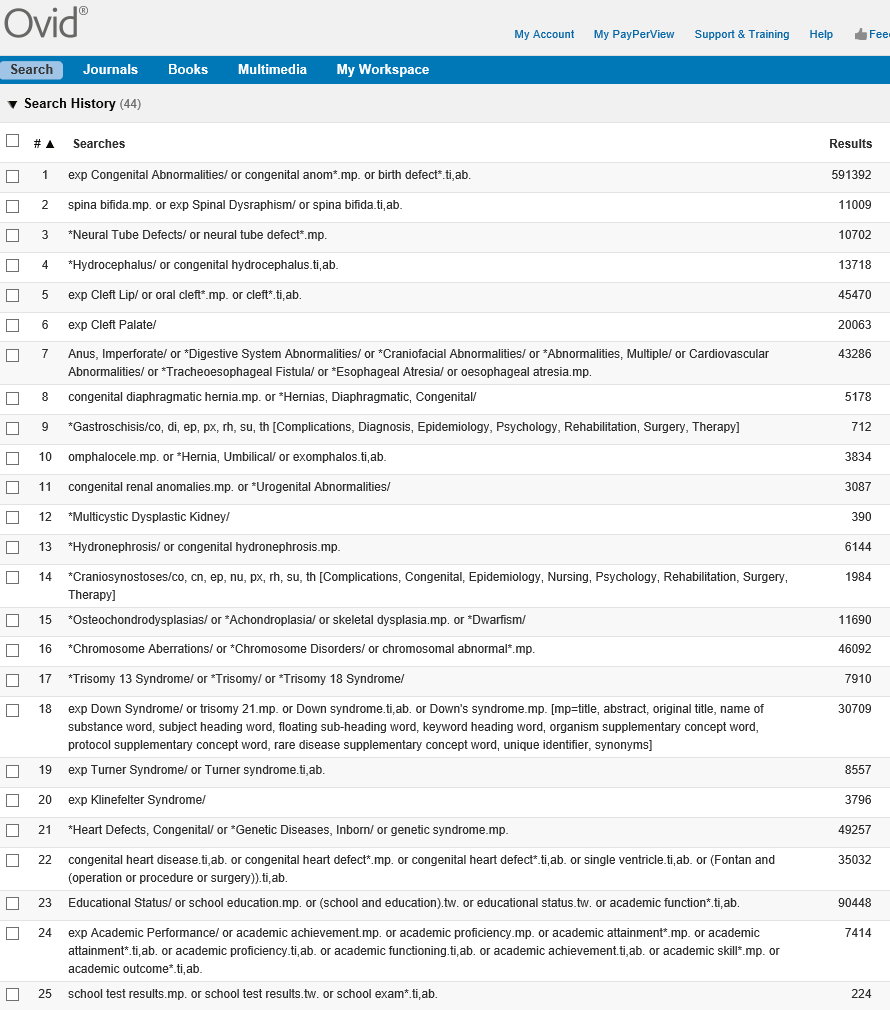


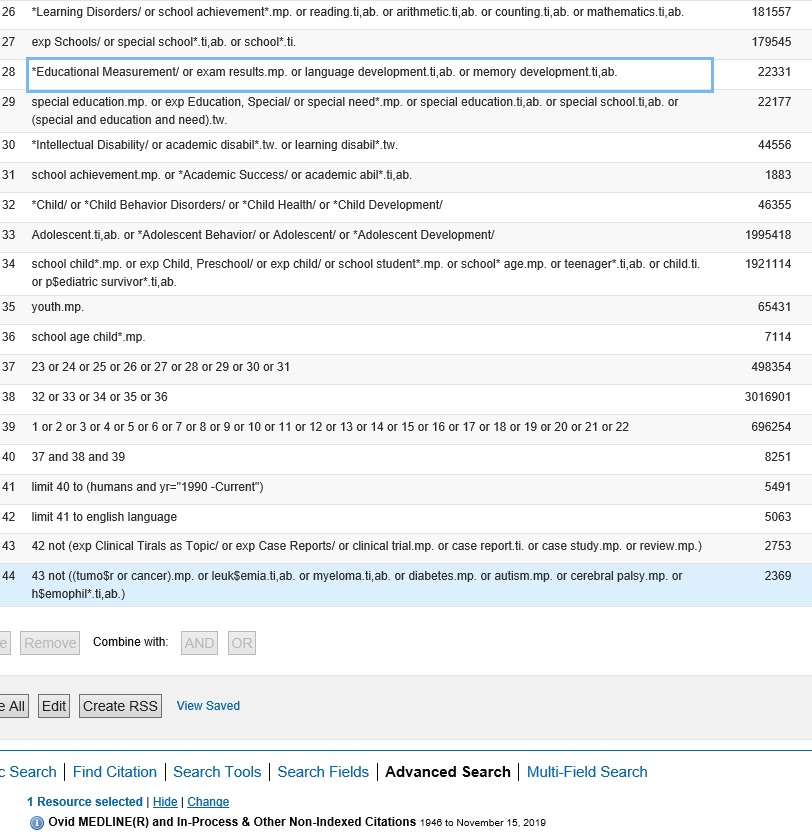


Medline (updated search on 01/12/2020)


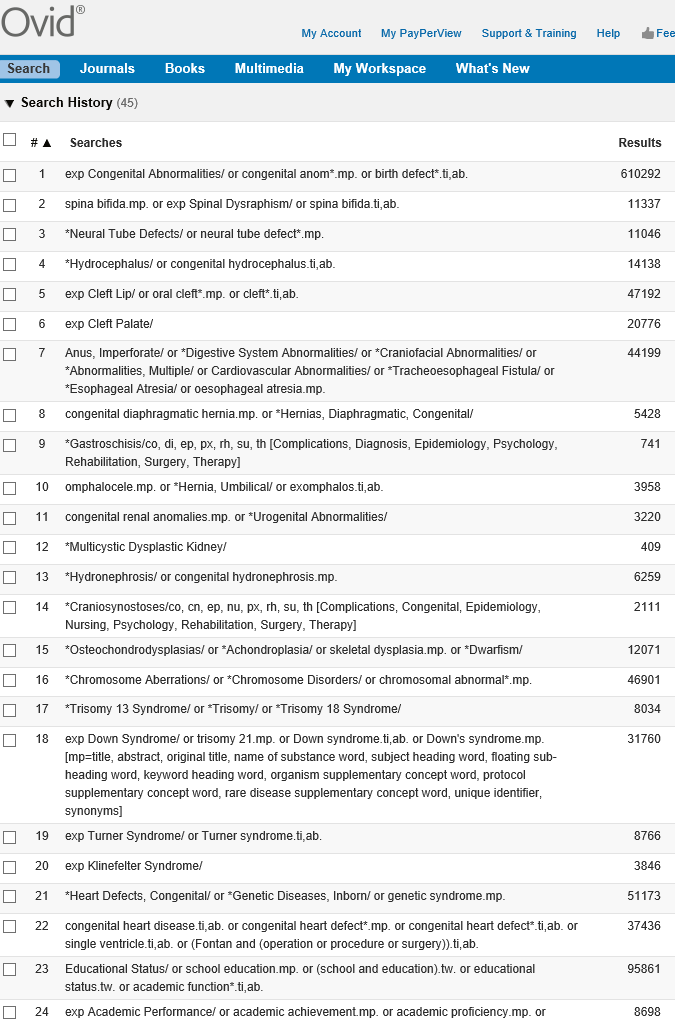


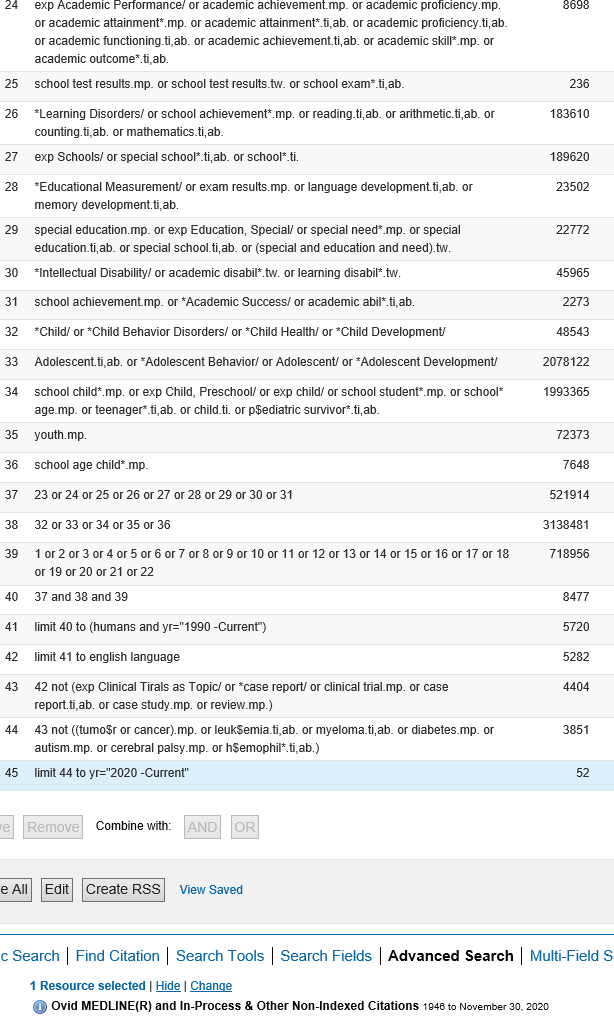


Embase (search on 18/11/2019)


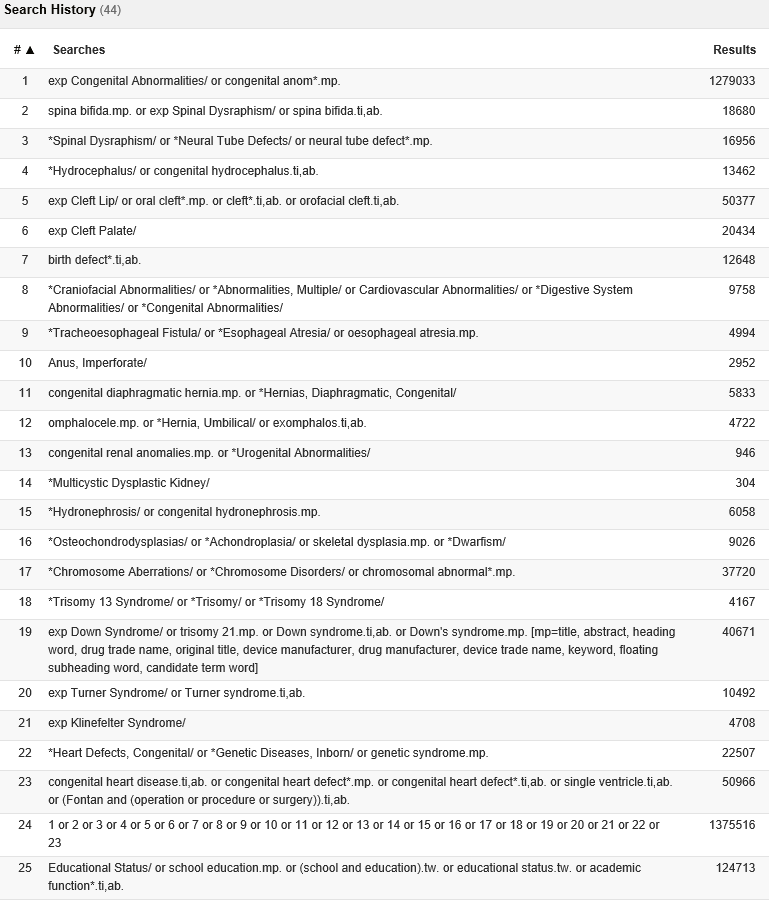


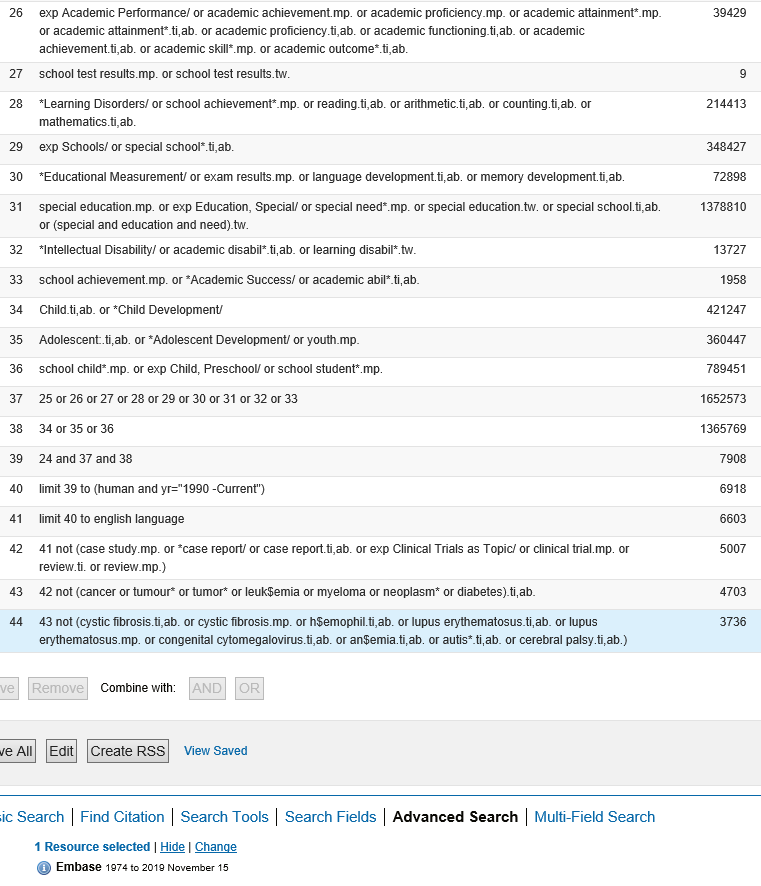


Embase (updated search on 01/12/2020)


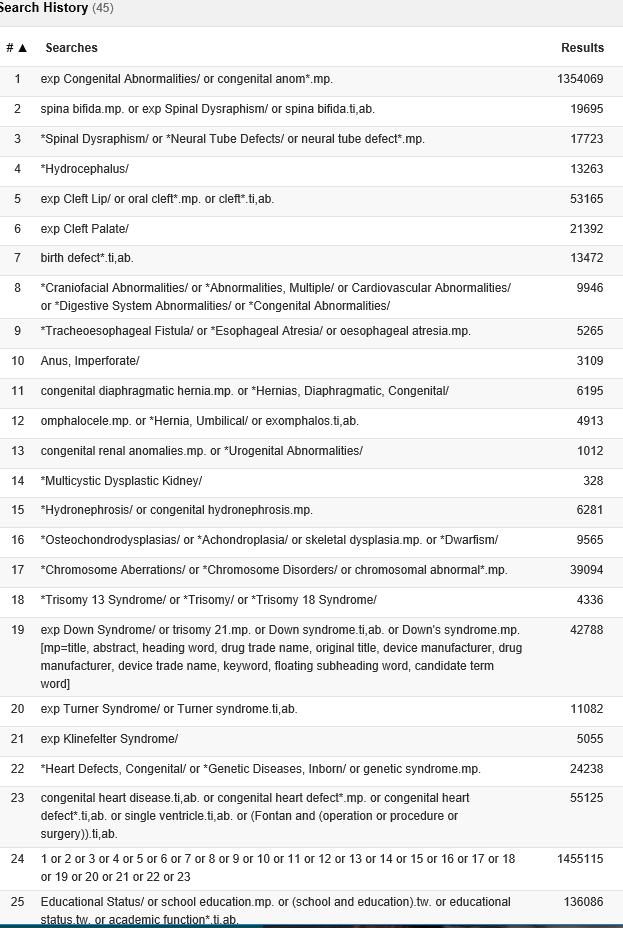


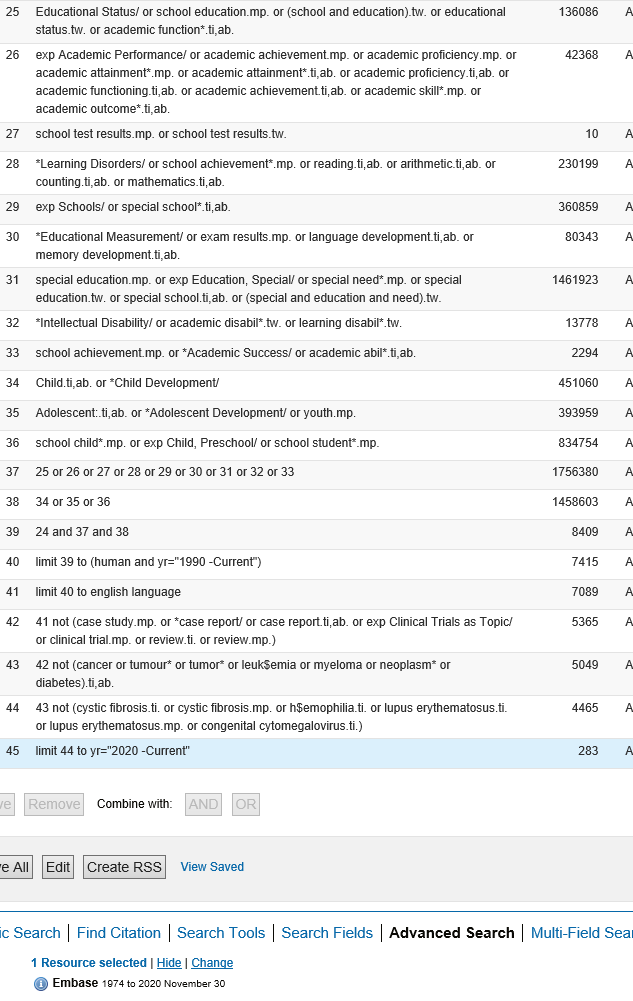

Supplement: Supplementary file 2 — TABLE S2 Search terms and search results in electronic databases Medline and Embase. [file BDR2-113-1431-s003.docx]
