# Supplementary material for: Academic achievement and needs of school‐aged children born with selected congenital anomalies: A systematic review and meta‐analysis
Source: Birth Defects Res. 2021 Oct 21;113(20):1431–62. doi: 10.1002/bdr2.1961 (PMC9298217; doi:10.1002/bdr2.1961)
Supplement: Supplementary file 3 — TABLE S3 Adapted Newcastle‐Ottawa Quality Assessment Scale for cohort and cross‐sectional studies. [file BDR2-113-1431-s007.docx]

TABLE S3 Adapted Newcastle-Ottawa Quality Assessment Scale for cohort and cross-sectional studies.

***NEWCASTLE - OTTAWA QUALITY ASSESSMENT SCALE***

***COHORT STUDIES***

Note: A study can be awarded a maximum of one star for each numbered item within the **Selection** and **Outcome** categories. A maximum of two stars can be given for **Comparability**

1) **Selection** (*max of 4 stars can be given – note: exposure is a diagnosis of a major congenital anomaly*)

Representativeness of the exposed cohort

a) truly representative of the average population of school aged children with a specific congenital anomaly in the community **🟑**

b) somewhat representative of the average population of school aged children with a specific congenital anomaly in the community **🟑**

c) selected group of users eg volunteers

d) no description of the derivation of the cohort

2) Selection of the non exposed cohort

a) drawn from the same community as the exposed cohort **🟑**

b) drawn from a different source

c) no description of the derivation of the non exposed cohort

3) Ascertainment of exposure

a) secure record (eg surgical records, register-based records, hospital records) **🟑**

b) written self report

c) no description

4) Demonstration that outcome of interest was not present at start of study

a) yes **🟑**

b) no

**Comparability** (*max of 2 stars can be given*)

1) Comparability of cohorts on the basis of the design or analysis

a) study controls for child’s age (select the most important factor) **🟑**

b) study controls for any additional factor**🟑** (This criteria could be modified to indicate specific control for a second important factor.)

c) no factors controlled for

**Outcome** (*max of 3 stars can be given – note: outcome is school test or standardised test results or special education needs*)

1) Assessment of outcome

a) independent blind assessment (standardised tests) **🟑**

b) record linkage with education databases**🟑**

c) self report

d) no description

2) Was follow-up long enough for outcomes to occur

a) yes (select an adequate follow up period for outcome of interest: children included in the analysis were the target age of the research question) **🟑**

b) no

3) Adequacy of follow up of cohorts

a) complete follow up - all subjects accounted for **🟑**

b) subjects lost to follow up unlikely to introduce bias - small number lost - > *80*% (select an adequate %) follow up, or description provided of those lost) **🟑**

c) follow up rate <_*80*% (select an adequate %) and no description of those lost

d) no statement

***NEWCASTLE - OTTAWA QUALITY ASSESSMENT SCALE***

***(adapted for cross sectional studies)***

1. **Selection:** (*Maximum 5 stars) (note: exposure is a diagnosis of a major congenital anomaly)*

1) Representativeness of the sample:

a) Truly representative of the average in the target population. **🟑** (all subjects or random sampling)

b) Somewhat representative of the average in the target population.**🟑** (non-random sampling)

c) Selected group of users.

d) No description of the sampling strategy.

2) Sample size:

a) Justified and satisfactory. **🟑**

b) Not justified.

3) Non-respondents:

a) Comparability between respondents and non-respondents characteristics is established, and the response rate is satisfactory. **🟑**

b) The response rate is unsatisfactory, or the comparability between respondents and non-respondents is unsatisfactory.

c) No description of the response rate or the characteristics of the responders and the non-responders.

4) Ascertainment of the exposure (risk factor - *note: exposure is a diagnosis of a major congenital anomaly)*:

a) Validated measurement tool (secure record, i.e. hospital record, surgical record or register-based). **🟑🟑**

b) Self-reported.

c) No description.

1. **Comparability:** (Maximum 2 stars)

1) The subjects in different outcome groups are comparable, based on the study design or analysis. Confounding factors are controlled.

a) The study controls for child’s age (select the most important factor). **🟑**

b) The study control for any additional factor (e.g. socioeconomic status). **🟑**

1. **Outcome:** (Maximum 3 stars)

1) Assessment of the outcome:

a) School test results or a reliable record of special education (via record linkage with educational databases). **🟑🟑**

b) Standardised achievement tests **🟑🟑**

c) Non-standardised test used or self report

d) No description.

2) Statistical test:

a) The statistical test used to analyze the data is clearly described and appropriate, and the measurement of the association is presented, including confidence intervals and the probability level (p value). **🟑**

b) The statistical test is not appropriate, not described or incomplete.
