# Supplementary material for: Academic achievement and needs of school‐aged children born with selected congenital anomalies: A systematic review and meta‐analysis
Source: Birth Defects Res. 2021 Oct 21;113(20):1431–62. doi: 10.1002/bdr2.1961 (PMC9298217; doi:10.1002/bdr2.1961)
Supplement: Supplementary file 4 — TABLE S4 Quality assessment scores of the included studies using the adapted versions of the Newcastle‐Ottawa scale for cohort and cross‐sectional studies. [file BDR2-113-1431-s002.docx]

TABLE S4 Quality assessment scores of the included studies using the adapted versions of the Newcastle-Ottawa scale for cohort and cross-sectional studies.

| **Reference, country** | **Selection** | **Comparability** | **Outcome** | **Category** |
| --- | --- | --- | --- | --- |
| **Cohort studies** |  |  |  |  |
| (Bell et al., 2017), Western Australia | **** | ** | *** | High |
| (Broder, Richman, & Matheson, 1998), USA | **** | * | *** | High |
| (Clausen et al., 2017). Denmark | **** | ** | *** | High |
| (Collett, Leroux, & Speltz, 2010), USA | **** | ** | ** | High |
| (Hentges et al., 2011), UK | **** | * | *** | High |
| (Hiraiwa et al., 2020), Japan | **** | * | *** | High |
| (Lawley et al., 2019) Australia | **** | * | *** | High |
| (Mlczoch et al., 2009), Austria | **** | * | *** | High |
| (Mulkey et al., 2016), USA | **** | * | *** | High |
| (Olsen et al., 2011), Denmark | **** | ** | *** | High |
| (Oster, Watkins, Hill, Knight, & Meyer, 2017), USA | **** | ** | *** | High |
| (Persson, Becker, & Svensson, 2012), Sweden | **** | ** | *** | High |
| (Riehle-Colarusso et al., 2015), USA | **** | ** | *** | High |
| (Speltz et al., 2015), USA | **** | ** | *** | High |
| (Speltz et al., 2017), USA & Canada | **** | ** | *** | High |
| (Watkins et al., 2018), USA | **** | * | *** | High |
| (Watkins et al., 2019), USA | **** | * | *** | High |
| (Wehby et al., 2014), USA | **** | ** | *** | High |
| (Wills, Holmbeck, Dillon, & McLone, 1990), USA | *** | * | *** | High |
| (Wotherspoon et al., 2020), Australia | **** |  | *** | High |
| Wray, 2001,(Wray & Sensky, 2001) UK | **** | ** | *** | High |
| (Wright & Nolan, 1994), Australia | **** |  | *** | High |
| (Yazdy, Autry, Honein, & Frias, 2008), USA | **** | ** | *** | High |
| **Cross-sectional studies** | |  |  |  |
| (Ayr, Yeates, & Enrile, 2005), USA | *** | ** | *** | High |
| (Barf et al., 2004), The Netherlands | ***** | ** | ** | High |
| (Barnes et al., 2006), Canada and USA | **** | ** | *** | High |
| (Bellinger et al., 2015), USA | *** |  | *** | Medium |
| (Chapman, 2011), USA | *** | * | *** | High |
| (Fitzsimons et al., 2018), England | ***** | * | *** | High |
| (Fitzsimons et al., 2021), England | ***** | * | *** | High |
| (Fletcher et al., 2005), Canada and USA | **** | * | *** | High |
| (Friedrich, Lovejoy, Shaffer, Shurtleff, & Beilke, 1991), USA | **** |  | *** | High |
| (Grewal et al., 2020), UK | ***** | * | *** | High |
| (Magge, Westerveld, Pruzinsky, & Persing, 2002), USA | *** |  | *** | Medium |
| (Mahle et al., 2000), USA | ***** | * | *** | High |
| (Saervold, Hide, Feragen, & Aukner, 2019), Norway | ***** |  | *** | High |
| (Sarrechia et al., 2016), Belgium | *** | ** | * | Medium |
| (Schaefer et al., 2016), Switzerland | **** |  | *** | High |
| (Simons, Glidden, Sheslow, & Pizarro, 2010), USA | **** | * | *** | High |

**Note:**

The Newcastle-Ottawa Quality Assessment Scale (NOS) for cohort studies (Wells et al.) (a maximum total 9 scores) and an amended version for cross-sectional studies (Modesti et al., 2016) (a maximum total 10 scores) were used to assess the quality of the included studies. Scores of <5 indicated high risk of bias (Luchini, Stubbs, Solmi, & Veronese, 2017).

A cohort study can be awarded a maximum of one star for each numbered item within the Selection (maximum 4 stars), Comparability (maximum 2 stars) and Outcome (maximum 3 stars) categories.

A cross-sectional study can be awarded a maximum of one star for each numbered item within the Selection, except for ascertainment of the exposure, i. e. diagnosis of a congenital anomaly type, where a maximum of 2 stars can be awarded (maximum 5 stars), Comparability (maximum 2 stars) and Outcome (maximum 3 stars) categories.

Quality categories were allocated as: Low = 0-2 stars, Medium = 3-5 stars, High = 6-9 stars (0-3 stars, 4-6 stars and 7-10 stars for cross-sectional studies respectively).

**References**

Ayr, L. K., Yeates, K. O., & Enrile, B. G. (2005). Arithmetic skills and their cognitive correlates in children with acquired and congenital brain disorder. *Journal of the International Neuropsychological Society, 11*, 249-262. doi:10.10170S1355617705050307

Barf, H. A., Verhoef, M., Post, M. W., Jennekens-Schinkel, A., Gooskens, R. H., Mullaart, R. A., & Prevo, A. J. (2004). Educational career and predictors of type of education in young adults with spina bifida. *International Journal of Rehabilitation Research, 27*, 45-52. doi:10.1097/00004356-200403000-00006

Barnes, M. A., Wilkinson, M., Khemani, E., Boudesquie, A., Dennis, M., & Fletcher, J. M. (2006). Arithmetic processing in children with spina bifida: Calculation accuracy, strategy use, and fact retrieval fluency. *Journal of Learning Disabilities, 39*, 174-187. doi:10.1177/00222194060390020601

Bell, J. C., Raynes-Greenow, C., Turner, R., Bower, C., Dodson, A., Nicholls, W., & Nassar, N. (2017). School performance for children with cleft lip and palate: a population-based study. *Child: Care, Health & Development, 43*, 222-231. doi:10.1111/cch.12388

Bellinger, D. C., Rivkin, M. J., DeMaso, D., Robertson, R. L., Stopp, C., Dunbar-Masterson, C., ... Newburger, J. W. (2015). Adolescents with tetralogy of Fallot: neuropsychological assessment and structural brain imaging. *Cardiology in the Young, 25*, 338-347. doi:10.1017/S1047951114000031

Broder, H. L., Richman, L. C., & Matheson, P. B. (1998). Learning disability, school achievement, and grade retention among children with cleft: a two-center study. *Cleft Palate-Craniofacial Journal, 35*, 127-131. doi:10.1597/1545-1569_1998_035_0127_ldsaag_2.3.co_2

Chapman, K. L. (2011). The relationship between early reading skills and speech and language performance in young children with cleft lip and palate. *Cleft Palate-Craniofacial Journal, 48*, 301-311. doi:10.1597/08-213

Clausen, N. G., Pedersen, D. A., Pedersen, J. K., Moller, S. E., Grosen, D., Wehby, G. L., ... Hansen, T. G. (2017). Oral Clefts and Academic Performance in Adolescence: The Impact of Anesthesia-Related Neurotoxicity, Timing of Surgery, and Type of Oral Clefts. *Cleft Palate-Craniofacial Journal, 54*, 371-380. doi:10.1597/15-185

Collett, B. R., Leroux, B., & Speltz, M. L. (2010). Language and early reading among children with orofacial clefts. *Cleft Palate-Craniofacial Journal, 47*, 284-292. doi:10.1597/08-172.1

Fitzsimons, K. J., Copley, L. P., Setakis, E., Charman, S. C., Deacon, S. A., Dearden, L., & van der Meulen, J. H. (2018). Early academic achievement in children with isolated clefts: a population-based study in England. *Archives of Disease in Childhood, 103*, 356-362. doi:10.1136/archdischild-2017-313777

Fitzsimons, K. J., Deacon, S. A., Copley, L. P., Park, M. H., Medina, J., & Van Der Meulen, J. H. (2021). School absence and achievement in children with isolated orofacial clefts. *Archives of Disease in Childhood, 106*, 154-159. doi:10.1136/archdischild-2020-319123

Fletcher, J. M., Copeland, K., Frederick, J. A., Blaser, S. E., Kramer, L. A., Northrup, H., ... Dennis, M. (2005). Spinal lesion level in spina bifida: a source of neural and cognitive heterogeneity. *Journal of Neurosurgery, 102*, 268-279. doi:10.3171/ped.2005.102.3.0268

Friedrich, W. N., Lovejoy, M. C., Shaffer, J., Shurtleff, D. B., & Beilke, R. L. (1991). Cognitive abilities and achievement status of children with myelomeningocele: a contemporary sample. *Journal of Pediatric Psychology, 16*, 423-428. doi:10.1093/jpepsy/16.4.423

Grewal, S. S., Ponduri, S., Leary, S. D., Wren, Y., Thompson, J. M. D., Ireland, A. J., ... Sandy, J. R. (2020). Educational Attainment of Children Born with Unilateral Cleft Lip and Palate in the United Kingdom. *Cleft Palate-Craniofacial Journal*, 1055665620959989. doi:10.1177/1055665620959989

Hentges, F., Hill, J., Bishop, D. V., Goodacre, T., Moss, T., & Murray, L. (2011). The effect of cleft lip on cognitive development in school-aged children: a paradigm for examining sensitive period effects. *Journal of Child Psychology & Psychiatry & Allied Disciplines, 52*, 704-712. doi:10.1111/j.1469-7610.2011.02375.x

Hiraiwa, A., Ibuki, K., Tanaka, T., Hirono, K., Miya, K., Yoshimura, N., & Ichida, F. (2020). Toddler neurodevelopmental outcomes are associated with school age IQ in children with single ventricle physiology. *Seminars in Thoracic and Cardiovascular Surgery, 32*, 302-310. doi:10.1053/j.semtcvs.2019.10.017

Lawley, C. M., Winlaw, D. S., Sholler, G. F., Martin, A., Badawi, N., Walker, K., ... Lain, S. J. (2019). School-Age Developmental and Educational Outcomes Following Cardiac Procedures in the First Year of Life: A Population-Based Record Linkage Study. *Pediatric Cardiology, 40*, 570-579. doi:10.1007/s00246-018-2029-y

Luchini, C., Stubbs, B., Solmi, M., & Veronese, N. (2017). Assessing the quality of studies in meta-analyses: Advantages and limitations of the Newcastle Ottawa Scale. *World Journal of Meta-Analysis, 5*, 80-84. doi:10.13105/wjma.v5.i4.80

Magge, S. N., Westerveld, M., Pruzinsky, T., & Persing, J. A. (2002). Long-term neuropsychological effects of sagittal craniosynostosis on child development. *Journal of Craniofacial Surgery, 13*, 99-104. doi:10.1097/00001665-200201000-00023

Mahle, W. T., Clancy, R. R., Moss, E. M., Gerdes, M., Jobes, D. R., & Wernovsky, G. (2000). Neurodevelopmental outcome and lifestyle assessment in school-aged and adolescent children with hypoplastic left heart syndrome. *Pediatrics, 105*, 1082-1089. doi:10.1542/peds.105.5.1082

Mlczoch, E., Albinni, S., Kitzmueller, E., Hanslik, A., Jalowetz, S., Male, C., & Salzer-Muhar, U. (2009). Special schooling in children with congenital heart disease: a risk factor for being disadvantaged in the world of employment. *Pediatric Cardiology, 30*, 905-910. doi:10.1007/s00246-009-9455-9

Modesti, P. A., Reboldi, G., Cappuccio, F. P., Agyemang, C., Remuzzi, G., Rapi, S., ... Settings, E. S. H. W. G. o. C. R. i. L. R. (2016). Panethnic Differences in Blood Pressure in Europe: A Systematic Review and Meta-Analysis. *PLoS One, 11*, e0147601. doi:10.1371/journal.pone.0147601

Mulkey, S. B., Bai, S., Luo, C., Cleavenger, J. E., Gibson, N., Holland, G., ... Bhutta, A. T. (2016). School-Age Test Proficiency and Special Education After Congenital Heart Disease Surgery in Infancy. *Journal of Pediatrics, 178*, 47-54. doi:10.1016/j.jpeds.2016.06.063

Olsen, M., Hjortdal, V. E., Mortensen, L. H., Christensen, T. D., Sorensen, H. T., & Pedersen, L. (2011). Educational achievement among long-term survivors of congenital heart defects: a Danish population-based follow-up study. *Cardiology in the Young, 21*, 197-203. doi:10.1017/S1047951110001769

Oster, M. E., Watkins, S., Hill, K. D., Knight, J. H., & Meyer, R. E. (2017). Academic Outcomes in Children With Congenital Heart Defects: A Population-Based Cohort Study. *Circulation: Cardiovascular Quality and Outcomes, 10*, e003074. doi:10.1161/CIRCOUTCOMES.116.003074

Persson, M., Becker, M., & Svensson, H. (2012). Academic achievement in individuals with cleft: a population-based register study. *Cleft Palate-Craniofacial Journal, 49*, 153-159. doi:10.1597/09-047

Riehle-Colarusso, T., Autry, A., Razzaghi, H., Boyle, C. A., Mahle, W. T., Van Naarden Braun, K., & Correa, A. (2015). Congenital Heart Defects and Receipt of Special Education Services. *Pediatrics, 136*, 496-504. doi:10.1542/peds.2015-0259

Saervold, T. K., Hide, O., Feragen, K. B., & Aukner, R. (2019). Associations Between Hypernasality, Intelligibility, and Language and Reading Skills in 10-Year-Old Children With a Palatal Cleft. *Cleft Palate-Craniofacial Journal, 56*, 1044-1051. doi:10.1177/1055665618824432

Sarrechia, I., Miatton, M., De Wolf, D., Francois, K., Gewillig, M., Meyns, B., & Vingerhoets, G. (2016). Neurocognitive development and behaviour in school-aged children after surgery for univentricular or biventricular congenital heart disease. *European Journal of Cardio-Thoracic Surgery, 49*, 167-174. doi:10.1093/ejcts/ezv029

Schaefer, C. J., Hoop, R., Schurch-Reith, S., Stambach, D., Kretschmar, O., Bauersfeld, U., ... Landolt, M. A. (2016). Academic achievement and satisfaction in adolescents with CHD. *Cardiology in the Young, 26*, 257-262. doi:10.1017/S1047951115000074

Simons, J. S., Glidden, R., Sheslow, D., & Pizarro, C. (2010). Intermediate neurodevelopmental outcome after repair of ventricular septal defect. *Annals of Thoracic Surgery, 90*, 1586-1591. doi:10.1016/j.athoracsur.2010.06.082

Speltz, M. L., Collett, B. R., Wallace, E. R., Starr, J. R., Cradock, M. M., Buono, L., ... Kapp-Simon, K. (2015). Intellectual and academic functioning of school-age children with single-suture craniosynostosis. *Pediatrics, 135*, e615-623. doi:10.1542/peds.2014-1634

Speltz, M. L., Wallace, E. R., Collett, B. R., Heike, C. L., Luquetti, D. V., & Werler, M. M. (2017). Intelligence and Academic Achievement of Adolescents with Craniofacial Microsomia. *Plastic & Reconstructive Surgery, 140*, 571-580. doi:10.1097/PRS.0000000000003584

Watkins, S. E., Allori, A. C., Meyer, R. E., Aylsworth, A. S., Marcus, J. R., & Strauss, R. P. (2019). Special education use in elementary school by children with nonsyndromic orofacial clefts. *Birth Defects Research, 111*, 142-150. doi:10.1002/bdr2.1418

Watkins, S. E., Meyer, R. E., Aylsworth, A. S., Marcus, J. R., Allori, A. C., Pimenta, L. A., ... Strauss, R. P. (2018). Academic achievement among children with nonsyndromic orofacial clefts: A population-based study. *Cleft Palate-Craniofacial Journal, 55*, 12-20. doi:10.1177/1055665617718823

Wehby, G. L., Collet, B., Barron, S., Romitti, P. A., Ansley, T. N., & Speltz, M. (2014). Academic achievement of children and adolescents with oral clefts. *Pediatrics, 133*, 785-792. doi:10.1542/peds.2013-3072

Wells, G. A., Shea, B., O’Connell, D., Peterson, J., Welch, V., Losos, M., & Tugwell, P. The Newcastle‐Ottawa Scale (NOS) for assessing the quality of non randomised studies in meta‐analyses. Retrieved from <http://www.ohri.ca/programs/clinical_epidemiology/oxford.asp>

Wills, K. E., Holmbeck, G. N., Dillon, K., & McLone, D. G. (1990). Intelligence and achievement in children with myelomeningocele. *Journal of Pediatric Psychology, 15*, 161-176. doi:10.1093/jpepsy/15.2.161

Wotherspoon, J. M., Eagleson, K. J., Gilmore, L., Auld, B., Hirst, A., Johnson, S., ... Justo, R. N. (2020). Neurodevelopmental and health-related quality-of-life outcomes in adolescence after surgery for congenital heart disease in infancy. *Developmental Medicine and Child Neurology, 62*, 214-220. doi:10.1111/dmcn.14251

Wray, J., & Sensky, T. (2001). Congenital heart disease and cardiac surgery in childhood: effects on cognitive function and academic ability. *Heart, 85*, 687-691. doi:10.1136/heart.85.6.687

Wright, M., & Nolan, T. (1994). Impact of cyanotic heart disease on school performance. *Archives of Disease in Childhood, 71*, 64-70. doi:10.1136/adc.71.1.64

Yazdy, M. M., Autry, A. R., Honein, M. A., & Frias, J. L. (2008). Use of special education services by children with orofacial clefts. *Birth Defects Research, 82*, 147-154. doi:10.1002/bdra.20433
