# Supplementary material for: Academic achievement and needs of school‐aged children born with selected congenital anomalies: A systematic review and meta‐analysis
Source: Birth Defects Res. 2021 Oct 21;113(20):1431–62. doi: 10.1002/bdr2.1961 (PMC9298217; doi:10.1002/bdr2.1961)
Supplement: Supplementary file 5 — TABLE S5 Studies reporting data on special education needs (SEN) or type of school for children born with specific congenital anomalies compared to the reference groups. [file BDR2-113-1431-s006.docx]

# TABLE S5 Studies reporting data on special education needs (SEN) or type of school for children born with specific congenital anomalies compared to the reference groups.

| **Author, publication year** | **Congenital anomaly (CA) type** | | **Patient age (years)**^†^ **or school grade/year** | **Cases, n** | **Special education needs (SEN) % (children with CAs)** | **SEN % (controls/general population)** | **Type of SEN, if reported** |
| --- | --- | --- | --- | --- | --- | --- | --- |
| ***Spina bifida*** | | | | | | | |
|  |  | | Primary school -4-11 yrs | n=118 | End of primary school: SEN - 59.3% (n=70); | Primary – 5% |  |
| Barf et al. (2004) | Spina bifida with hydrocephalus | | secondary – ≥12 years | n=118 | Completed special secondary school: 42.4% (n=50); did not complete secondary school 8.5% (n=10) | Secondary – 11% | Not reported |
| ***Congenital heart defects (CHD)*** | | | | | | | |
| Hiraiwa et al. (2020) | CHD - single ventricle physiology | | 8 years of age | n=35 | 28.6% (n=10), no *p* value reported. | 3.1% of public school students | Not reported |
| Lawley et al. (2019) | CHD with a surgery during the first year of life | | First year of primary school (7 years) | n=260 | 13.1% (n=34), *p*<0.05 compared to the reference population | 4.4% in those without a cardiac surgery in the first year of life (n=261,915) | Not reported |
| Mahle et al. (2000) | Hypoplastic left heart syndrome (HLHS) | | 9.0 ± 2.1 years | n=115 | 32.2% (n=37) of children received some form of special education: 100% - 9.6%; >50%, but <100% - 7.8%, <50% - 14.7% | 9-12% in the included US studies | Not reported |
| Mlczoch et al. (2009) | CHD (86% after cardiac surgery) | | Median 12 years (range 6-19) | n=227 | 15% (95% CI 10-20), *p*<0.001 | 3.6% in the reference population | Not reported |
| Mulkey et al. (2016) | CHD with a surgery at age <1 year | | Grade 3-4: with known SEN status | n=334 | 26.9% (n=90), *p* <0.001 | 11.6% (n=55,020) in the grade-matched students (n=476,083) | Intellectual disability (ID): 5.4% vs 1.2% (ref); specific learning disability (SLD): 6.0% vs 3.8%; multiple disabilities: 2.7% vs 0.3% |
| Oster et al. (2017) | CHD | | 3rd grade (9 years): | n=2803 | 20.5% (aOR^‡^=1.64 (95% CI 1.44-1.86), critical CHD: 25.1%, non-critical: 19.6% | 12.5% in controls without CAs (n=6341) | Not reported |
| Riehle-Colarusso et al. (2015) | CHD: critical and non-critical | | 3-10 years of age, linked to special education files (1992-2012 school years) | Any CHD (3744): critical CHD (n=843), noncritical CHD (2901) | 14.9% (n=558) for any CHD (per survivors at 3 years of age): aPRR§=1.5 (95% CI 1.4–1.7); critical: 16.7% (n=141), non-critical: 12.75% (n=417) | 9.2% (n=79,141) in the reference cohort (860,715) | Any ID: aPRR§3.8 (95% CI 2.8, 5.1); sensory impairment: aPRR§ 3.0 (95% CI 1.8, 5.0); significant developmental delay: aPRR§ 1.9 (95% CI 1.3, 2.8); SLD: aPRR§ 1.4 (95% CI 1.1, 1.7); other health impairment: aPRR§ 2.8 (95% CI 2.2, 3.5) |
| Sarrechia et al. (2016) | CHD: UVH (univentricular) vs controls and vs BiVH (biventricular) | | UVH: 9.1 (±2.1) years/months, range: 6-12 years; BiVH: 9.0 (±2.2) | UVH=17, BiVH=46 | UVH: 11.8% (*p*=0.485 vs controls by Fisher exact test); BiVH: 8.7% | 0% in matched controls (n=17) | Not reported |
| Wright et al. (1994) | Cyanotic CHD (surgery at <2.5 years) vs controls | | Mean 9.5 (±1.2) y, range (7-11.4 y), (n=29) vs controls (n=36) | n=29 | Referred for extra assistance at school 27.6% (n=8), RR=2.48 (0.81-7.58), *p*=0.114 | 11.1% (n=4) in controls (n=36) | Not reported |
| ***Orofacial clefts (OFCs)*** | | |  |  |  |  |  |
| Collett et al. (2010) | | Non-syndromic cleft lip & palate (CLP) and cleft palate (CP) | 7 years old | CLP (n=23) | 56.5% (n=13) | 19.3% (n=11) in controls (n=57) | Communication disability: 85% of all with SEN |
|  | | |  | CP (n=22) | 40.9% (n=9) |  | Communication disability 44% and specific learning disability 33% of all with SEN |
| Fitzsimons et al. (2018) | Isolated OFCs | | 5 years old between 2006 and 2012 | All OFC (n=2769) | 29.4% (n=815) | 9.7% national rate for 5-year olds at the same time period | SEN type in 565 with documented SEN: Speech, language and communication needs (SLCN) 73.6%, Learning difficulties 13.6%, Behavior, emotional and social; difficulties 9.2%, Hearing impairment 7.6%, Other 14.3%. |
|  |  | |  | Cleft lip (CL) | 13.2% |  |  |
|  |  | |  | CP | 33.1% |  |  |
|  |  | |  | Unilateral CLP | 34.1% |  |  |
|  |  | |  | Bilateral CLP | 47.6% |  |  |
| Fitzsimons et al. (2021) | Isolated OFCs | | 7 years old | All OFC (n=3517) | 40.5% (n=1426) | 20.9% national rate | Most common: SLCN:14.5% vs national rate of 2.5% (SEN type reported for 63.1% of those with SEN (n=900, with SLCN 70.2% (n=510)) |
|  |  | |  | CL | 26.7% |  |  |
|  |  | |  | CP | 42.8% |  |  |
|  |  | |  | CLP | 47.9% |  |  |
| Hentges et al. (2011) | Isolated CL with/without CP | | Mean=7.7 (±0.64) years | n=79¶ with SEN data | 31.6% (n=25), *p*<0.001 vs controls | 8.5% (n=6) in controls (n=71¶) | Not reported |
|  |  | |  | Early surgery (n=37) | 27.0% (n=10), *p*=0.03 vs controls |  |  |
|  |  | |  | Late surgery (n=42) | 35.7% (n=15), *p*<0.001 vs controls |  |  |
| Watkins et al. (2019) | Nonsyndromic OFCs | | 3^rd^ grade of elementary school | n=523 | 35.4%, (n=185) PR 3.02 (95% CI 2.50, 3.64) vs controls (reference) | 14.9% (n=961) in controls (n=6448) | Excluding speech and language type: PR 1.86 (95% CI 1.45, 2.38) |
|  |  | | 4^th^ grade | n=439 | 35.1%, PR 2.67 (95% CI 2.19, 3.26) | 15.5% |  |
|  |  | | 5^th^ grade | n=356 | 28.9%, RP 2.01 (95% CI 1.60, 2.52) | 15.5% |  |
| Wehby et al. (2014) | Isolated OFCs | | Grades 2-11 | n=2,300 | 19.7% (n=453), *p*<0.01 in regression models compared to controls | 12.9% (n=1018) in controls (n=7865) | Not reported |
| Yazdy et al. (2008) | OFCs | | Age range 3-10 years | All with OFC (n=777) | At least 1 year in special education: 25.9% (n=201), PR=3.2 (95% CI: 2.9–3.6) vs controls | 8% (n=59,136) in controls (n=737,528) | For all OFC: most frequent services used SCLN (81.1%): PR 3.8 (95% CI 3.3, 4.3) compared to controls; after excluding children with only SLCN, PR 2.4 (95% CI 1.7, 3.2), |
|  |  | |  | Isolated OFC (n=645): | 22.9% (n=148), PR=2.9 (95% CI: 2.5–3.3) vs controls |  | for isolated OFC: PR 1.6 (95% CI 1.0, 2.4). |
|  |  | |  | CP (n=239) | 23.4% (n=56) |  |  |
|  |  | |  | CL (n=126) | 13.5% (n=17) |  |  |
|  |  | |  | CLP (n=280) | 26.8% (n=75) |  |  |

†Mean (±SD) or median (IQR) or range.

‡ Adjusted for maternal education, race/ ethnicity, public pre-Kindergarten enrolment, and gestational age.

§ Adjusted for maternal age at delivery, race/ethnicity, maternal education, infant gender, birth weight, and birth year group.

¶ The number of children with known SEN status for cases and controls was calculated by authors of this review and approved by authors of the original paper (Hentges et al. (2011).

aPRR, adjusted prevalence rate ratio; BiVH, biventricular heart defect; CA, congenital anomaly; HLHS, hypoplastic left heart syndrome; ID, intellectual disability; OFC, orofacial cleft; PR, prevalence ratio; SLCN, Speech, language and communication needs; SLD, specific learning disability; UVH, univentricular heart defect (HLHS/tricuspid atresia).

**References**

Barf, H. A., Verhoef, M., Post, M. W., Jennekens-Schinkel, A., Gooskens, R. H., Mullaart, R. A., & Prevo, A. J. (2004). Educational career and predictors of type of education in young adults with spina bifida. *International Journal of Rehabilitation Research, 27*, 45-52. doi:10.1097/00004356-200403000-00006

Collett, B. R., Leroux, B., & Speltz, M. L. (2010). Language and early reading among children with orofacial clefts. *Cleft Palate-Craniofacial Journal, 47*, 284-292. doi:10.1597/08-172.1

Fitzsimons, K. J., Copley, L. P., Setakis, E., Charman, S. C., Deacon, S. A., Dearden, L., & van der Meulen, J. H. (2018). Early academic achievement in children with isolated clefts: a population-based study in England. *Archives of Disease in Childhood, 103*, 356-362. doi:10.1136/archdischild-2017-313777

Fitzsimons, K. J., Deacon, S. A., Copley, L. P., Park, M. H., Medina, J., & Van Der Meulen, J. H. (2021). School absence and achievement in children with isolated orofacial clefts. *Archives of Disease in Childhood, 106*, 154-159. doi:10.1136/archdischild-2020-319123

Hentges, F., Hill, J., Bishop, D. V., Goodacre, T., Moss, T., & Murray, L. (2011). The effect of cleft lip on cognitive development in school-aged children: a paradigm for examining sensitive period effects. *Journal of Child Psychology & Psychiatry & Allied Disciplines, 52*, 704-712. doi:10.1111/j.1469-7610.2011.02375.x

Hiraiwa, A., Ibuki, K., Tanaka, T., Hirono, K., Miya, K., Yoshimura, N., & Ichida, F. (2020). Toddler neurodevelopmental outcomes are associated with school age IQ in children with single ventricle physiology. *Seminars in Thoracic and Cardiovascular Surgery, 32*, 302-310. doi:10.1053/j.semtcvs.2019.10.017

Lawley, C. M., Winlaw, D. S., Sholler, G. F., Martin, A., Badawi, N., Walker, K., . . . Lain, S. J. (2019). School-Age Developmental and Educational Outcomes Following Cardiac Procedures in the First Year of Life: A Population-Based Record Linkage Study. *Pediatric Cardiology, 40*, 570-579. doi:10.1007/s00246-018-2029-y

Mahle, W. T., Clancy, R. R., Moss, E. M., Gerdes, M., Jobes, D. R., & Wernovsky, G. (2000). Neurodevelopmental outcome and lifestyle assessment in school-aged and adolescent children with hypoplastic left heart syndrome. *Pediatrics, 105*, 1082-1089. doi:10.1542/peds.105.5.1082

Mlczoch, E., Albinni, S., Kitzmueller, E., Hanslik, A., Jalowetz, S., Male, C., & Salzer-Muhar, U. (2009). Special schooling in children with congenital heart disease: a risk factor for being disadvantaged in the world of employment. *Pediatric Cardiology, 30*, 905-910. doi:10.1007/s00246-009-9455-9

Mulkey, S. B., Bai, S., Luo, C., Cleavenger, J. E., Gibson, N., Holland, G., . . . Bhutta, A. T. (2016). School-Age Test Proficiency and Special Education After Congenital Heart Disease Surgery in Infancy. *Journal of Pediatrics, 178*, 47-54. doi:10.1016/j.jpeds.2016.06.063

Oster, M. E., Watkins, S., Hill, K. D., Knight, J. H., & Meyer, R. E. (2017). Academic Outcomes in Children With Congenital Heart Defects: A Population-Based Cohort Study. *Circulation: Cardiovascular Quality and Outcomes, 10*, e003074. doi:10.1161/CIRCOUTCOMES.116.003074

Riehle-Colarusso, T., Autry, A., Razzaghi, H., Boyle, C. A., Mahle, W. T., Van Naarden Braun, K., & Correa, A. (2015). Congenital Heart Defects and Receipt of Special Education Services. *Pediatrics, 136*, 496-504. doi:10.1542/peds.2015-0259

Sarrechia, I., Miatton, M., De Wolf, D., Francois, K., Gewillig, M., Meyns, B., & Vingerhoets, G. (2016). Neurocognitive development and behaviour in school-aged children after surgery for univentricular or biventricular congenital heart disease. *European Journal of Cardio-Thoracic Surgery, 49*, 167-174. doi:10.1093/ejcts/ezv029

Watkins, S. E., Allori, A. C., Meyer, R. E., Aylsworth, A. S., Marcus, J. R., & Strauss, R. P. (2019). Special education use in elementary school by children with nonsyndromic orofacial clefts. *Birth Defects Research, 111*, 142-150. doi:10.1002/bdr2.1418

Wehby, G. L., Collet, B., Barron, S., Romitti, P. A., Ansley, T. N., & Speltz, M. (2014). Academic achievement of children and adolescents with oral clefts. *Pediatrics, 133*, 785-792. doi:10.1542/peds.2013-3072

Wright, M., & Nolan, T. (1994). Impact of cyanotic heart disease on school performance. *Archives of Disease in Childhood, 71*, 64-70. doi:10.1136/adc.71.1.64

Yazdy, M. M., Autry, A. R., Honein, M. A., & Frias, J. L. (2008). Use of special education services by children with orofacial clefts. *Birth Defects Research, 82*, 147-154. doi:10.1002/bdra.20433
