# Supplementary material for: Academic achievement and needs of school‐aged children born with selected congenital anomalies: A systematic review and meta‐analysis
Source: Birth Defects Res. 2021 Oct 21;113(20):1431–62. doi: 10.1002/bdr2.1961 (PMC9298217; doi:10.1002/bdr2.1961)
Supplement: Supplementary file 6 — TABLE S6 Risk factors associated with poorer academic performance and/or special education needs (SEN) in children with specific congenital anomalies (CAs). [file BDR2-113-1431-s005.docx]

# TABLE S6 Risk factors associated with poorer academic performance and/or special education needs (SEN) in children with specific congenital anomalies (CAs)

| **Author, publication year** | **Assessed area of learning** | **Risk factor group** | **Unadjusted OR/RR/PRR/z-score (95% CI)** | **Adjusted OR/RR/PRR/z-score (95% CI)** | **Factors adjusted for** |
| --- | --- | --- | --- | --- | --- |
| ***Spina bifida*** | |  |  |  |  |
| Barf et al., 2004 | Special secondary education (% vs regular) | Shunted for hydrocephalus (HC) (Yes vs No (ref gr) | 92% vs 51%, *p*<0.001 | OR 4.43 (95% CI 1.4, 13.3), *p*<0.05 | IQ<85, ambulation (wheelchair dependent), continence, level of lesion, type of SB, annual number of surgical interventions |
|  |  | IQ below average (≤85) vs IQ>85 | 71% vs 26%, *p*<0.001 | OR 5.1 (95% CI 2.4, 11.0), *p*<0.001 | Presence of HC, ambulation (wheelchair dependent), continence, level of lesion, type of SB, annual number of surgical interventions |
|  |  | Ambulation (wheelchair dependent) | 75% vs 33%, *p*<0.001 | OR 2.6 (95% CI 1.1, 6.0), *p*<0.05 | IQ<85, presence of HC, continence, level of lesion, type of SB, annual number of surgical interventions |
|  |  | Type of spina bifida (SB) - aperta | 97% vs 69%, *p*<0.001 | Dropped from the multivariable model as NS |  |
|  |  | Level of lesion - L2 and higher | 62% vs 29%, *p*<0.001 | Dropped from the multivariable model as NS |  |
|  |  | Continence - incontinent | 80% vs 62%, *p*<0.01 | Dropped from the multivariable model as NS |  |
|  |  | Annual number of surgical interventions - >0.33 | 68% vs 40%, *p*<0.001 | Dropped from the multivariable model as NS |  |
| Wills et al., 1990 | Maths scores | Age across 4-12 years (continuous) | Negative correlation with increasing age: *r*=─0.40, *p*<0.01 | NA | NA |
|  | Reading scores | Age across 4-12 years | NS correlation with age: *r*=─0.11 |  |  |
|  | Spelling scores | Age across 4-12 years | NS correlation with age: *r*=─0.11 |  |  |
| Fletcher et al., 2005 |  | Ethnicity and lesion level | Mean (±SD) | NA | NA |
|  | WJTA-R: basic reading | Hispanic - upper lesions | 71.4 (±28.5), *p*<0.02 for lesion level, *p*<0.0001 for ethnicity |  |  |
|  |  | Hispanic - lower lesions | 84.9 (±27.8) |  |  |
|  |  | Non-Hispanic - upper lesions | 91.3 (±22.6) |  |  |
|  |  | Non-Hispanic lower lesions | 96.1 (±19.9) |  |  |
|  | WJTA-R: passage comprehension | Hispanic - upper lesions | 62.0 (±26.5), *p*<0.0002 for lesion level, *p*<0.0001 for ethnicity |  |  |
|  |  | Hispanic - lower lesions | 81.2 (±29.3) |  |  |
|  |  | Non-Hispanic - upper lesions | 88.6 (±19.6) |  |  |
|  |  | Non-Hispanic lower lesions | 95.9 (±18.7) |  |  |
|  | WJTA-R: calculations | Hispanic - upper lesions | 56.5 (±30.8), *p*<0.0001 for lesion level, *p*<0.008 for ethnicity |  |  |
|  |  | Hispanic - lower lesions | 79.9 (±28.0) |  |  |
|  |  | Non-Hispanic - upper lesions | 74.6 (±23.9) |  |  |
|  |  | Non-Hispanic lower lesions | 80.3 (±24.0) |  |  |
| ***Congenital heart defects (CHDs)*** | | |  |  |  |
| Bellinger et al., 2015 | WIAT Mathematics composite in cases with Tetralogy Fallot without genetic anomalies (n=68) | ≥2 complications at first surgery vs none | NA | β estimate ±SE: −29.1 ± 6.8 (*p*<0.001, linear regression) | Concurrent family social status |
|  | WIAT Reading | Seizures (vs none) |  | −18.4 ± 6.9 (*p*=0.01) | Concurrent family social status |
|  | WIAT Reading | Genetic diagnosis | NA | 82.5 (±24.1) vs 96.1 (±17.8) in those without genetic diagnosis *(p*=0.003), vs population mean *p*=0.002 | Concurrent family social status |
|  | WIAT Maths | Genetic diagnosis |  | 73.1 (±27.1) vs 95.1 (±25.6) without genetic diagnosis *(p*<0.001), vs population mean *(p*<0.001 | Concurrent family social status |
| Hiraiwa et al., 2020 | Special education services | Mental developmental index at age 3 years <85 vs ≥85 | 53.3% vs 10%, *p*=0.0082 | NA | NA |
|  |  | Later age at surgical stage III (Fontan procedure) | OR 1.31 (1.02, 1.69), *p*=0.0075 |  |  |
|  |  |  | OR (95% CI) | aOR (95% CI) |  |
| Lawley et al., 2019 | Performing below the national minimum standard in: Reading | Low birth weight (LBW, <2500g) vs reference (≥2500g) | 2.79 (1.26, 6.17) | 1.86 (0.75, 4.61) | Parental education, student language at home, total number of readmission to age 6 |
|  |  | Parental education below 12 years/not stated vs year12 or equivalent | 3.07 (1.62, 5.81) | 2.79 (1.39-, .63) | Birth weight, student language at home, total number of readmissions to age 6 |
|  |  | Middle Eastern or Central Asian language at home vs English | 3.18 (1.29, 7.86) | 3.31 (1.19, 9.20) | Birth weight, parental education, total number of readmissions to age 6 |
|  |  | Multiple readmissions to hospital before 6 years of age vs 0 (reference): 5-7 | 2.30 (0.45, 10.67) | 4.68 (1.33, 16.43) | Birth weight, parental education, student language at home |
|  |  | >7 | 11.77 (3.45, 40.10) | 12.81 (3.93, 41.77) |  |
|  | Numeracy | LBW vs reference (≥2500g) | 4.32 (2.07, 9.01) | 3.07 (1.35, 6.96) | Parental education, student language at home, total number of readmissions to age 6, length of stay during the initial procedure admission |
|  |  | Parental education below 12 years/not stated vs year12 or equivalent | 2.74 (1.52, 4.98) | 2.41 (1.26, 4.58) | Birth weight, student language at home, total number of readmissions to age 6, length of stay during the initial procedure admission |
|  |  | Middle Eastern or Central Asian language at home vs English | 3.20 (1.34, 7.65) | 3.23 (1.21, 8.58) | Birth weight, parental education, total number of readmissions to age 6, length of stay during the initial procedure admission |
|  |  | Multiple readmissions to hospital before 6 years of age vs 0 (reference): 5-7 | 1.21 (0.37, 3.99) | 2.00 (0.68, 5.90) | Birth weight, parental education, student language at home, length of stay during the initial procedure admission |
|  |  | >7 | 4.32 (1.78, 10.48) | 3.83 (1.38, 10.60) |  |
|  |  | Length of stay during the initial procedure admission - ≥21 days | 4.72 (1.47, 15.12) | 2.71 (0.77, 9.51) | Birth weight, parental education, student language at home, total number of readmissions to age 6 |
|  | Writing | LBW vs reference (≥2500g) | 5.29 (2.51, 11.16) | 3.92 (1.67, 9.23) | Parental education, student language at home, total number of readmissions to age 6, length of stay during the initial procedure admission |
|  |  | Parental education below 12 years/not stated vs year12 or equivalent | 3.89 (1.98, 7.62) | 3.21 (1.55, 6.63) | Birth weight, student language at home, total number of readmissions to age 6, length of stay during the initial procedure admission |
|  |  | Middle Eastern or Central Asian language at home vs English | 2.22 (0.84, 5.90) | NS | Birth weight, parental education, total number of readmissions to age 6, length of stay during the initial procedure admission |
|  |  | Multiple readmissions to hospital before 6 years of age vs 0 (reference): 5-7 | 1.75 (0.24, 12.75) | 7.51 (1.89, 30.15) | Birth weight, parental education, student language at home, length of stay during the initial procedure admission |
|  |  | >7 | 16.73 (3.86, 72.49) | 16.19 (4.20, 62.39) |  |
|  |  | Length of stay during the initial procedure admission - ≥21 days | 3.59 (1.22, 10.55) | 1.56 (0.37, 6.54) | Birth weight, parental education, student language at home, total number of readmissions to age 6 |
| Mahle et al., 2000 | Scores on WJTA-Math | Longer cumulative cardiopulmonary bypass (CPB) | Lower scores *(p* =0.02) | NA | NA |
|  | Scores on WJTA-Reading | Longer cumulative CPB | Lower scores *(p* =0.007) | NA | NA |
| Mulkey et al., 2016 | Not achieving grade proficiency in literacy† | Longer duration of hospitalization (75^th^ quartile 24 d) vs 25^th^ (8 d – ref) | NA | OR 0.7 (0.45, 0.96), *p*=0.03 | The following variables we entered into the models: 5-minute Apgar score, maternal education; meal status at school, distance to hospital at birth, |
|  |  | Lower 5-minute Apgar score | NA | OR 0.6 (0.41, 0.99), *p*=0.04 | total surgical time, CHD diagnosis, CPB, age at surgery, sex, race, grade level, duration of hospitalization. |
|  |  | Lower maternal education (75^th^ quartile 14 y) vs 25^th^ (12 – ref) | NA | OR 1.3 (1.01, 1.67), *p*=0.04 |  |
|  |  | Receipt of free meal at school (full price vs free meal (ref)) | NA | OR 2.7 (1.36, 5.40), *p*=0.02 |  |
|  |  | Closer distance to hospital at birth (75^th^ quartile -138 miles) vs 25^th^ (17miles – ref) | NA | OR 1.8 (1.09, 3.05), *p*=0.02 |  |
|  | Not achieving grade proficiency in mathematics† | Non-white race (white vs non-white (ref) | NA | OR 3.0 (1.59, 5.8), *p*=0.001 | The same variables as listed above |
|  |  | Use of CPB (Yes vs No (ref)) | NA | OR 0.4 (0.2, 0.998), *p*=0.049 |  |
|  |  | Longer duration of hospitalization (75^th^ quartile 24 d) vs 25^th^ (8 d – ref) | NA | OR 0.7 (0.45, 0.96), *p*=0.03 |  |
|  |  | Receipt of free-meal at school (full price vs free meal (ref)) | NA | OR 2.6 (1.26, 5.26), *p*=0.01 |  |
|  | Receipt of special education† | Longer duration of hospitalization (75^th^ quartile -24 d) vs (25^th^ -7 d – ref) | NA | OR 1.95 (1.44, 4.29), *p*=0.001 | The same variables as listed above |
| Olsen et al., 2011 | Completed compulsory basic schooling | After exclusion of preterm or with extracardiac or chromosomal anomalies (n=2260) | NA | aHR 0.87 (95% CI 0.83, 0.92) vs reference cohort | Current age, sex, parental income, number of siblings, having a single parent, and parents’ highest educational level |
| Oster et al., 2017 | End of third-grade tests: Reading (not meeting standards) | CHD severity: critical CHD and non-critical CHD vs controls | Critical CHD: 40.8% vs 31.3% (controls); non-critical CHD - 39.8% vs 31.3% (controls). | Critical CHD: aOR 1.59 (1.29, 1.96); non-critical CHD: aOR 1.34 (1.19, 1.50) | Maternal education, race/ethnicity, and public pre-Kindergarten enrolment |
|  | End of third-grade tests: Maths (not meeting standards) | CHD severity: critical CHD and non-critical CHD vs controls | Critical CHD - 28.1% vs 21.1% (controls); non-critical CHD - 25.0% vs 21.1% (controls). | Critical CHD: aOR 1.48 (1.18, 1.86); non-critical CHD: aOR1.07 (0.95, 1.22) | Maternal education, race/ethnicity, and public pre-Kindergarten enrolment |
|  |  | critical vs non-critical CHD | 28.1% vs 25.0% | aOR 1.38 (1.08, 1.75) | Maternal education, race/ethnicity, and public pre-Kindergarten enrolment |
|  | Third-grade retention | CHD severity: critical CHD and non-critical CHD vs controls | Critical CHD - 2.8% vs 2.0% (controls); non-critical CHD - 2.8% vs 2.0% (controls). | Critical CHD: aOR 1.44 (0.80, 2.60); non-critical CHD: aOR 1.29 (0.93, 1.80) | Maternal education, race/ethnicity, and public pre-Kindergarten enrolment |
|  | SEN (Exceptional services) | CHD severity: critical CHD and non-critical CHD vs controls | Critical CHD - 25.1% vs 12.5% (controls); non-critical CHD - 19.6% vs 12.5% (controls). | Critical CHD: aOR 2.24 (1.79, 2.81); non-critical CHD: aOR 1.51 (1.37, 1.73) | Maternal education, race/ethnicity, and public pre-Kindergarten enrolment |
|  |  | critical vs non-critical CHD | 25.1% vs 19.6% | aOR 1.46 (1.15, 1.86) | Maternal education, race/ethnicity, and public pre-Kindergarten enrolment |
|  | End of third-grade tests: Reading (not meeting standards) | Presence of additional anomalies: with other noncardiac vs isolated CHD | 44.3% vs 38.8% | aOR 1.31 (1.07, 1.59) | Maternal education, race/ethnicity, and public pre-Kindergarten enrolment |
|  | End of third-grade tests: Maths (not meeting standards) | Presence of additional anomalies: with other noncardiac vs isolated CHD | 28.7% vs 24.7% | aOR 1.24 (1.00, 1.54) | Maternal education, race/ethnicity, and public pre-Kindergarten enrolment |
|  | End of third-grade tests: both Reading and Maths | Presence of additional anomalies: with other noncardiac vs isolated CHD | 24.7% vs 19.8% | aOR 1.36 (1.08, 1.71) | Maternal education, race/ethnicity, and public pre-Kindergarten enrolment |
|  | Third-grade retention | Presence of additional anomalies: with other noncardiac vs isolated CHD | 3.4% vs 2.7% | aOR 1.24 (0.73, 2.11) | Maternal education, race/ethnicity, and public pre-Kindergarten enrolment |
|  | SEN (Exceptional services) | Presence of additional anomalies: with other noncardiac vs isolated CHD | 28.7% vs 18.3% | aOR 1.72 (1.39, 2.14) | Maternal education, race/ethnicity, and public pre-Kindergarten enrolment |
| Riehle-Colarusso et al., 2015 | Special education services: other health impairment | CHD severity: critical | 47.4% | aPRR 5.4 (95% CI 3.8, 7.7) vs reference population | Adjusted for maternal age at delivery, race/ethnicity, education, infant gender, birth weight, and birth year group |
|  |  | non-critical | 18.6% | 2.0 (95% CI 1.5, 2.8) vs reference population | Adjusted for maternal age at delivery, race/ethnicity, education, infant gender, birth weight, and birth year group |
|  | Other SEN types | CHD severity |  | NS for any other SEN types |  |
| Schaefer et al., 2016 | Level of secondary education I (7-9 grades) | CHD severity: severe | Low level: 23.7% vs 9.9% in controls, *p*=0.03 | OR 2.15 (0.59, 7.82), *p*=0.25 | SES, foreign language, open heart surgery, cyanotic CHD |
|  |  | Lower socioeconomic status (SES) | NA | OR 1.39 (0.25,-7.70), *p*=0.71 | Severity of CHD, foreign language, open heart surgery, cyanotic CHD |
|  |  | Foreign language | NA | OR 0.80 (0.20, 3.17), *p*=0.75 | SES, severity of CHD, open heart surgery, cyanotic CHD |
|  |  | Cyanotic CHD | NA | OR 1.15 (0.31, 4.37), *p*=0.84 | SES, foreign language, severity of CHD, open heart surgery |
|  |  | Open heart surgery | NA | OR 1.46 (0.52, 4.07), *p*=0.47 | SES, foreign language, severity of CHD, cyanotic CHD |
| ***Craniofacial anomalies - Orofacial clefts (OFCs)*** | | |  |  |  |
| Bell et al., 2017 | Meeting national minimum standards: Reading | Type of OFC (CL, CP, CLP) vs children without OFC (reference group) | WALNA: CL: OR 0.89 (0.50, 1.59); CLP: 1.07 (0.61, 1.88); CP: 1.13 (0.72, 1.78). NAPLAN: CL - OR 0.91 (0.47, 1.77); CLP: 0.74 (0.39, 1.41); CP: 0.63 (0.40, 0.99). | WALNA: CL: OR 1.13 (0.63, 2.04); CLP:1.29 (0.72, 2.32); CP: 0.88 (0.56, 1.39). NAPLAN: CL: OR 1.04 (0.51, 2,11); CLP: 1.04 (0.48, 2.26); CP: 0.57 (0.34, 0.96). | WALNA: OFC type, school year level, Indigenous background (yes/no), socioeconomic quartile, child's relative age; the same for NAPLAN + family order. |
|  | Numeracy | Type of OFC (CL, CP, CLP) vs children without OFC (reference group) | WALNA: CL: OR 0.75 (0.47, 1.20); CLP: 0.66 (0.43, 1.00); CP: 0.72 (0.49, 1.06). NAPLAN: CL: OR 0.74 (0.36, 1.52); CLP: 0.52 (0.28, 0.99); CP: 0.58 (0.35, 0.96). | WALNA: CL: OR 0.84 (0.51, 1.37); CLP: 0.71 (0.46, 1.11); CP: 0.64 (0.43, 0.95). NAPLAN: CL: OR 0.75 (0.36, 1.56); CLP: 0.73 (0.34, 1.57); CP: 0.47 (0.28, 0.82). | WALNA: OFC type, school year level, Indigenous background (yes/no), socioeconomic quartile, the same for NAPLAN + school location. |
|  | Writing | Type of OFC (CL, CP, CLP) vs children without OFC (reference group) | WALNA: CL: OR 0.70 (0.46, 1.08); CLP: 0.79 (0.52, 1.18); CP: 0.81 (0.58, 1.14). NAPLAN: CL: OR 0.66 (0.38, 1.15); CLP: 0.49 (0.29, 0.84); CP: 0.75 (0.48, 1.10). | WALNA: CL: OR 0.83 (0.53, 1.30); CLP: 0.88 (0.55, 1.39); CP: 0.66 (0.47, 0.92). NAPLAN: CL: OR 0.77 (0.39, 1.49); CLP: 0.77 (0.37, 1.59); CP: 0.62 (0.38, 1.00). | WALNA: OFC type, school year level, Indigenous background (yes/no), socioeconomic quartile, the same for NAPLAN + family order + birthweight. |
|  | Spelling | Type of OFC (CL, CP, CLP) vs children without OFC (reference group) | WALNA: CL: OR 0.78 (0.47, 1.30); CLP: 0.74 (0.46, 1.19); CP: 1.07 (0.70, 1.65). NAPLAN: CL: OR 0.78 (0.43, 1.43); CLP: 0.41 (0.25, 0.69); CP: 0.81 (0.53, 1.23). | WALNA: CL: OR 0.89 (0.50, 1.62); CLP: 0.88 (0.52, 1.48); CP: 0.94 (0.60, 1.46). NAPLAN: CL: OR 0.96 (0.49, 1.88); CLP: 0.52 (0.29, 0.94); CPO: 0.68(0.42, 1.10). | WALNA: OFC type, school year level, Indigenous background (yes/no), socioeconomic quartile, the same for NAPLAN + family order + school location + language background not English. |
|  | Reading | Presence of additional anomalies‡ (isolated OFC – ref group) | 5 year (NAPLAN): OR 0.39 (0.14, 1.07); 9 year (WALNA): OR 0.22 (0.04, 1.32) | NA | NA |
|  | Numeracy | With additional anomalies vs isolated | NAPLAN: 3 year: OR 0.40 (0.11, 1.31); 5 year: OR 0.34 (0.09, 1.25); | NA | NA |
|  | Writing | With additional anomalies vs isolated | 5 year (NAPLAN): OR 3.62 (0.46, 28.58); 5 year (WALNA): OR 2.35 (0.64, 8.59); 7 year (combined years): OR 1.84 (0.83, 4.08) | NA | NA |
|  | Spelling | With additional anomalies vs isolated | 3 year (NAPLAN): OR 0.30 (0.10, 0.88); | NA | NA |
| Broder et al., 1998 | Learning disability | Child's sex and cleft type | CP: males 79%, females 42%; CLP: males 37%, females 47% (significant interaction between sex and cleft type, *p*=0.004) | NA | NA |
|  | Percentage below grade-level performance on achievement tests | Child's sex and cleft type | CP: males 62.5%, females 42%; CLP: males 43%, females 50% (NS). | NA | NA |
|  | Prevalence of grade retention | Child's sex and cleft type | CP: males 38%, females 19%; CLP: males 24%, females 32% (significant interaction between sex and cleft type: *p*=0.002) | NA | NA |
| Chapman et al., 2011 | TERA-3 (reading quotient) | Delayed vs normal speech | Mean 93.53 (±10.91) vs 105.38 (±10.91), (*t*=2.87, *p*=0.008) | NA | NA |
| Clausen et al., 2017 | 9^th^ Grade exam† | OFC type | Mean test score difference (95% CI) | Mean test score difference (95% CI) | Sex, birth weight, paternal/maternal age and level of parental education. |
|  |  | CL vs controls | 0.12 (−0.06, 0.29) | 0.12 (−0.05, 0.29) |  |
|  |  | CLP vs controls | −0.15 (−0.31, 0.01) | -0.06 (−0.21, 0.09) |  |
|  |  | CP vs controls | −0.13 (−0.32, 0.06) | -0.20 (−0.38, −0.03) |  |
|  |  | Sex (ref boys) |  |  | Cleft type, birth weight, paternal/ maternal age and level of education. |
|  |  | Girls | 0.31 (0.27, 0.34) | 0.35 (0.31, 0.38) |  |
|  |  | Maternal age |  |  | Sex, birth weight, paternal age, cleft type and level of parental education. |
|  |  | (<20) vs 20-27 years | –0.56 (–0.68, –0.44) | –0.19 (–0.31, –0.08) |  |
|  |  | 28-35 | 0.30 (0.26, 0.34) | 0.08 (0.04, 0.13) |  |
|  |  | ≥36 | 0.45 (0.37, 0.53) | 0.14 (0.05, 0.23) |  |
|  |  | Maternal education (ref – basic school) |  |  | Sex, maternal/paternal age, cleft type, birth weight, and level of paternal education |
|  |  | Vocational | 0.31 (0.26, 0.36) | 0.24 (0.19, 0.29) |  |
|  |  | Short§ | 0.84 (0.79, 0.88) | 0.58 (0,.52, 0.63) |  |
|  |  | Long¶ | 1.37 (1.28, 1.46) | 0.83 (0.73, 0.93) |  |
|  | Nonattainment at final exam† | OFC type | OR (95% CI) | OR (95% CI) | Sex, birth weight, parental age and level of parental education. |
|  |  | CL vs controls | 0.86 (0.53, 1.39) | 0.79 (0.46, 1.35) |  |
|  |  | CLP vs controls | 1.22 (0.84, 1.77) | 1.07 (0.71, 1.61) |  |
|  |  | CP vs controls | 2.54 (1.83, 3.52) | 2.59 (1.78, 3.76) |  |
|  |  | Sex (ref boys) |  |  | Cleft type, birth weight, paternal/ maternal age and level of education. |
|  |  | Girls | 0.59 (0.53, 0.65) | 0.55 (0.50, 0.62) |  |
|  |  | Maternal age (years) |  |  | Sex, birth weight, paternal age, cleft type and level of parental education |
|  |  | (<20) vs 20-27 | 1.90 (1.50, 2.41) | 1.34 (1.01, 1.79) |  |
|  |  | 28-35 | 0.75 (0.67, 0.83) | 0.90 (0.79, 1.03) |  |
|  |  | Maternal education (ref – basic school) |  |  | Sex, maternal/paternal age, cleft type, birth weight, and level of paternal education |
|  |  | Vocational | 0.54 (0.48, 0.60) | 0.59 (0.52, 0.67) |  |
|  |  | Short§ | 0.38 (0.33, 0.43) | 0.50 (0.43, 0.59) |  |
|  |  | Long¶ | 0.26 (0.18, 0.36) | 0.47 (0.32, 0.69) |  |
|  |  | Birth weight in grams (ref - 3000-3999g) |  |  | Sex, maternal/paternal age, cleft type and level of parental education |
|  |  | ≤1499 | 2.84 (1.78, 4.54) | 2.93 (1.74, 4.94) |  |
|  |  | 1500-1999 | 2.33 (1.58, 3.44) | 1.95 (1.23, 3.09) |  |
|  |  | 2000-2499 | 1.58 (1.23, 2.02) | 1.33 (1.00, 1.76) |  |
|  |  | 2500-2999 | 1.35 (1.18, 1.54) | 1.25 (1.07, 1.45) |  |
|  | Average test scores | Number of cleft operations | NS associations with test scores in patients of either sex with any OFC type | NA |  |
| Collett et al., 2010 | WJTA-R reading composite | CLP vs control | NA | β ±SE: 5.74±4.65 | Sex, race/ethnicity, SES, age at assessment, mother’s marital status, mother’s work hours, mother’s age, number of siblings |
|  |  | CP vs control | NA | β ±SE: 10.27±4.73 (*p*=0.03) |  |
|  | Special education | CLP vs control | NA | aOR = 3.8 (*p*=0.02) | The same demographic factors |
|  |  | CP vs control | NA | aOR 3.6 (*p*=0.07) |  |
| Fitzsimons et al., 2018 |  | Cleft type: CL - reference group |  | Difference in z-scores compared with CL with 95% CI (multivariable linear regression) |  |
|  | Communication, language and literacy (reading and writing) | CP | NA | ─0.28 (─0.38, 0.19) | Two measures of economic deprivation (IDACI, area-based index, and free school meal eligibility, sex and ethnicity |
|  |  | Unilateral CLP | NA | ─0.19 (─0.28, 0.09) |  |
|  |  | Bilateral CLP | NA | ─0.30 (─0.43, 0.17) |  |
|  | Mathematical development | CP | NA | ─0.27 (─0.37, 0.17) | The same factors as above |
|  |  | Unilateral CLP | NA | 0.15 (─0.26, 0.05) |  |
|  |  | Bilateral CLP | NA | ─0.19 (─0.33, 0.04) |  |
|  | Communication, language and literacy | SES (area-based) – 1 (most deprived) |  | reference | Cleft type, free school meal eligibility (measure of economic deprivation), sex and ethnicity |
|  |  | 2 |  | 0.11 (0.00, 0.22) |  |
|  |  | 3 |  | 0.32 (0.21, 0.44) |  |
|  |  | 4 |  | 0.48 (0.36, 0.59) |  |
|  |  | 5 (least deprived) |  | 0.58 (0.46, 0.71) |  |
|  | Mathematical development | SES (area-based) – 1 (most deprived) |  | reference | The same factors as above |
|  |  | 2 |  | 0.18 (0.07, 0.30) |  |
|  |  | 3 |  | 0.40 (0.28, 0.53) |  |
|  |  | 4 |  | 0.55 (0.42, 0.68) |  |
|  |  | 5 (least deprived) |  | 0.71 (0.57, 0.84) |  |
|  | Communication, language and literacy | Free school meals - No |  | reference | Cleft type, IDACI quintile (area-based index of economic deprivation), sex and ethnicity |
|  |  | Yes |  | ─0.47 (─0.57, 0.38) |  |
|  | Mathematical development | Free school meals - No |  | reference | The same factors as above |
|  |  | Yes |  | ─0.41 (─0.52, 0.31) |  |
| Fitzsimons et al., 2021 | Achieving expected level (≥2) in composite education outcome (5 subjects) | Cleft type: CL (reference group) | **Model 1:** OR=1 | **Model 2:** OR=1 | Model 1: adjusted for school absence only, Model 2: plus sex, IDACI quintile and free school meal eligibility |
|  |  | CP | OR 0.72 (0.60, 0.88) | OR 0.64 (0.52, 0.78) |  |
|  |  | CLP | OR 0.76 (0.63, 0.92) | OR 0.77 (0.63, 0.94) |  |
|  | Achieving expected level (≥2) in composite education outcome (5 subjects) | School absence: 0-5 days | 77.4% (reference OR=1) | 1 | Adjusted for cleft type, sex, IDACI quintile and free school meal eligibility |
|  |  | 5/5-10 days | 71.6% (OR 0.73 (0.60, 0.89)) | OR 0.75 (0.61, 0.92) |  |
|  |  | 10.5-15 days | 66.6% (OR 0.58 (0.47, 0.72)) | OR 0.63 (0.50, 0.79) |  |
|  |  | 15.5-20 days | 59.1% (OR 0.42 (0.33, 0.54)) | OR 0.52 (0.40, 0.68) |  |
|  |  | ≥20 days | 43.4% (OR 0.22 (0.18, 0.28)) | OR 0.30 (0.23, 0.38) |  |
| Grewal et al., 2020 | School tests: average point score | Significant functional variables: decayed missing filled teeth (DMFT) :1-3 vs 0 (ref)) | **Model 1**: coef (95% CI)=─1.02 (─2.09, 0.05) | **Model 2:** coef (95% CI)= ─0.70 (─1.74, 0.34) | Model 1: birth month and sex; model 2: birth month, sex and SES |
|  | (linear regression) | 4+ | ─1.89 (─3.08, ─0.71), *p*=0.001 | ─1.40 (─2.59, ─0.20), *p*=0.02 |  |
|  |  | Intelligibility: different but intelligible vs normal (ref) | ─0.45 (─1.54, 0.64) | ─0.40 (─1.45, 0.65) |  |
|  |  | Just intelligible or less | ─2.97 (─4.29, ─1.65), *p*<0.001 | ─2.44 (─3.72, ─1.16), *p*=0.001 |  |
|  |  | Psychological: low self-confidence | ─1.99 (─3.80, ─1.72), *p*=0.03 | ─1.60 (─3.36, 0.16), *p*=0.08 |  |
|  |  | Minimum number of problems: 2 vs 0-1 (ref) | ─0.70 (─1.70, 0.29) | ─0.49 (─1.45, 0.47) |  |
|  |  | 3-6 | ─2.67 (─3.96, ─1.38)*, p*<0.001 | ─2.26 (─3.55, ─0.97)*, p*=0.002 |  |
|  | Reading: achieving expected level (≥2) (logistic regression) | DMFT: 1-3 vs 0 (ref)) | Model 1: aOR 0.48 (0.18, 1.27) | Model 2: aOR 0.53 (0.20, 1.45) | Model 1: birth month and sex; model 2: birth month, sex and SES |
|  |  | 4+ | aOR 0.28 (0.11, 0.74), *p*=0.009 | aOR 0.36 (0.13, 1.00), *p*=0.05 |  |
|  |  | Hearing loss: Any vs normal hearing | aOR 0.38 (0.14, 0.97), *p*=0.04 | aOR 0.38 (0.14, 1.00), *p*=0.05 |  |
|  |  | Intelligibility: different but intelligible vs normal (ref) | aOR 0.81 (0.25, 2.60) | aOR 0.82 (0.25, 2.67) |  |
|  |  | Just intelligible or less | aOR 0.17 (0.06, 0.48), *p*=0.002 | aOR 0.20 (0.07, 0.59), *p*=0.01 |  |
|  |  | Minimum number of problems: 2 vs 0-1 (ref) | aOR 0.64 (0.24, 1.67) | aOR 0.73 (0.27, 1.96) |  |
|  |  | 3-6 | aOR 0.20 (0.07, 0.53), *p*=0.002 | aOR 0.23 (0.08, 0.65), *p*=0.01 |  |
|  | Writing: achieving expected level (≥2) | DMFT: 1-3 vs 0 (ref)) | Model 1: aOR 0.49 (0.21, 1.19) | Model 2: aOR 0.55 (0.23, 1.34) | Model 1: birth month and sex; model 2: birth month, sex and SES |
|  |  | 4+ | aOR 0.35 (0.14, 0.86), *p*=0.02 | aOR 0.44 (0.17, 1.13), *p*=0.08 |  |
|  |  | Intelligibility: different but intelligible vs normal (ref) | aOR 0.62 (0.24, 1.58) | aOR 0.62 (0.24, 1.61) |  |
|  |  | Just intelligible or less | aOR 0.34 (0.12, 0.92), *p*=0.03 | aOR 0.42 (0.15, 1.18), *p*=0.09 |  |
|  |  | Minimum number of problems: 2 vs 0-1 (ref) | aOR 0.76 (0.33, 1.75) | aOR 0.86 (0.37, 2.02) |  |
|  |  | 3-6 | aOR 0.36 (0.14, 0.92), *p*=0.04 | aOR 0.43 (0.17, 1.13), *p*=0.1 |  |
| Saervold et al., 2019 | Reading measured by Word Chain test | Presence of hypernasality | 6.1 (±1.77) vs 6.4 (±1.56) for normal, *p*>0.05 | NA | NA |
|  | Reading measured by Reading Comprehension test |  | 4.8 (±1.67) vs 5.2 (±1.64), *p*>0.05 | NA | NA |
|  | Reading measured by Word Chain test | Intelligibility (normal vs reduced) | 6.4 (±1.46 vs 5.9 (±2.25), *p*>0.05 | NA | NA |
|  | Reading measured by Reading Comprehension test |  | 5.4 (±1.53) vs 3.9 (±1.75), *p*<0.01, effect size=0.91 | NA | NA |
| Watkins et al., 2018 | Not meeting standards in end of 3rd-grade tests: Reading | Type of OFC vs children without CA (reference group): CL (n=139) | NA | aOR 1.32 (0.97, 1.92) | Maternal education, race/ethnicity, and public pre-Kindergarten enrolment |
|  |  | CP (n=159) |  | aOR 1.02 (0.71, 1.47) |  |
|  |  | CLP (n=188) |  | aOR 1.33 (0.96, 1.83) |  |
|  | Mathematics | CL (n=140) | NA | aOR 1.28 (0.49, 1.31) | Maternal education, race/ethnicity, and public pre-Kindergarten enrolment |
|  |  | CP (n=159) |  | aOR 1.23 (0.84, 1.88) |  |
|  |  | CLP (n=189) |  | aOR 1.20 (0.97, 1.98) |  |
|  | Both Reading and Maths | CL (n=139) | NA | aOR 0.86 (0.49, 1.51) | Maternal education, race/ethnicity, and public pre-Kindergarten enrolment |
|  |  | CP (n=159) |  | aOR 1.14 (0.71, 1.83) |  |
|  |  | CLP (n=188) |  | aOR 1.74 (1.19, 2.56) |  |
| Watkins, 2019 | Special education | Cleft type vs controls |  |  |  |
|  | 3^rd^ grade†† | CL | PR 1.06 (0.67, 1.67) | NA | NA |
|  |  | CP | PR 3.44 (2.55, 4.65) | NA | NA |
|  |  | CLP | PR 4.61 (3.49, 6.09) | NA | NA |
|  | Special education |  |  |  |  |
|  | 3^rd^ grade | Race/Ethnicity in OFCs vs controls |  | NA | NA |
|  |  | White | PR 3.04 (2.42, 3.81) | NA | NA |
|  |  | African American | PR 2.94 (1.85, 4.67) | NA | NA |
|  |  | Hispanic | PR 3.72 (1.98, 7.01) | NA | NA |
|  |  | Other | PR 2.33 (0.93, 5.86) | NA | NA |
| Wehby et al., 2014 |  | Cleft type: | β ±SE from basic regression (differences with matched classmates) | β ±SE from expanded regression specification | In basic regression - adjusted for grade, form (of test) years (since test version had |
|  | Reading | CL | −4.07±2.16 (*p*<0.1) | −3.61±2.06 (*p*<0.1) | been implemented & group (that the child was in) |
|  |  | CP | −6.24±2.7 (*p*<0.05) | −6.42±2.51 (*p*<0.05) |  |
|  |  | CLP | −2.41±1.98 (*p*≥0.1) | −1.66±1.84 (*p*≥0.1) | In expanded regression – the same |
|  | Language | CL | −5.06±2.13 (*p*<0.05) | −4.35±2.02 (*p* <0.05) | variables as in basic regression plus |
|  |  | CP | −6.24±2.64 (*p* <0.05) | −5.95±2.54 (*p* <0.05) | maternal age, marital status at child’s |
|  |  | CLP | −4.26±1.92 (*p* <0.05) | −3.57±1.80 (*p* <0.05) | birth, race/ethnicity, education, any |
|  | Mathematics | CL | −4.20±1.94 (*p* <0.05) | −3.42±1.80 (*p*<0.1) | smoking and any alcohol consumption |
|  |  | CP | −6.33±2.48 (*p* <0.05) | −6.26±2.39 (*p* <0.01) | anytime during pregnancy, and father’s |
|  |  | CLP | −4.97±1.79 (*p* <0.01) | −4.20±1.69 (*p* <0.05) | education and age at child’s birth. |
|  | Composite Total | CL | −5.20±2.43 (*p* <0.05) | −3.90±2.27 (*p* <0.1) |  |
|  |  | CP | −6.61±2.80 (*p* <0.05) | −7.01±2.56 (*p* <0.01) |  |
|  |  | CLP | −3.47±2.13 (*p* ≥0.1) | −2.62±1.99 (*p* ≥0.1) |  |
|  | Reading, Language. or Maths <25^th^ percentile | CL | 0.071±0.031 (*p* <0.05) | 0.060±0.029 (*p* <0.05) |  |
|  |  | CP | 0.115±0.040 (*p* <0.01) | 0.115±0.039 (*p* <0.01) |  |
|  |  | CLP | 0.059±0.029 (*p* <0.05) | 0.050±0.028 (*p* <0.1) |  |
|  | Special education | CL | 0.035±0.032 (*p* <0.1) | 0.019±0.030 (*p* <0.1) |  |
|  |  | CP | 0.126±0.040 (*p* <0.01) | 0.117±0.039 (*p* <0.01) |  |
|  |  | CLP | 0.093±0.032 (*p* <0.01) | 0.088±0.030 (*p* <0.01) |  |
|  | Reading, Language. or Maths <25^th^ percentile (OFC) | School level: elementary school | 0.080±0.020 (*p*<0.01) |  |  |
|  |  | Middle school | 0.078±0.024 (*p*<0.01) |  |  |
|  |  | High school | 0.067±0.026 (*p*<0.05) |  |  |
|  | Special education (OFC) | Elementary school | 0.075±0.023 (*p*<0.01) |  |  |
|  |  | Middle school | 0.089±0.026 (*p*<0.01) |  |  |
|  |  | High school | 0.070±0.025 (*p*<0.01) |  |  |
| Yazdy et al., 2008 | Special education | Cleft type: CL | PR 1.7 (95% CI 1.1–2.6) vs controls | NA | NA |
|  |  | CP | PR 2.9 (95% CI 2.3–3.7) vs controls |  |  |
|  |  | CLP | PR 3.3 (95% CI 2.8–4.1) vs controls |  |  |
|  | Special education | Maternal race/ethnicity: NHW: Isolated OFC | PR 2.4 (95% CI 2.0, 2.9) vs ref (NHW without CAs) |  |  |
|  |  | NHB: Isolated OFC | PR 3.4 (95% CI 2.6, 4.3) vs ref (NHB without CAs) |  |  |
|  |  | Hispanic: Isolated OFC | PR 7.1 (95% CI 4.5, 11.0) vs ref (Hispanic without CAs); 29.8% vs 20.2% in NHW with isolated OFC |  |  |
|  | Special education | Maternal education: 0-12 years: Isolated OFC | PR 3.3 (95% CI 2.8, 3.8) vs ref (0-12 yrs for those without CAs) |  |  |
|  |  | >12 years: Isolated OFC | PR 3.1 (95% CI 2.6, 3.8) vs ref (>12 yrs for those without CAs) |  |  |
| ***Other craniofacial anomalies - Craniosynostosis*** | | |  |  |  |
| Speltz et al., 2015 |  | Type of cranio-synostosis (each vs sagittal) |  |  |  |
|  | WRAT reading | Metopic | Not reported | -3.0 (-8.4, 2.4), *p*= 0.27 | Age, gender, SES and maternal IQ |
|  |  | Unicoronal |  | -11.7 (-16.8, -6.7), *p*<0.001 | Age, gender, SES and maternal IQ |
|  |  | Lambdoid |  | 14.8 (-25.8, -3.7), *p*=0.009 | Age, gender, SES and maternal IQ |
|  | WRAT spelling | Metopic |  | -2.9 (-7.9, 2.2), *p*=0.26 | Age, gender, SES and maternal IQ |
|  |  | Unicoronal |  | -10.1 (-15.4, -4.8), *p*<0.001 | Age, gender, SES and maternal IQ |
|  |  | Lambdoid |  | -10.8 (-22.2, 0.6), *p*=0.06 | Age, gender, SES and maternal IQ |
|  | WRAT Math computation | Metopic |  | -1.8 (-6.3, 2.7), *p*=0.44 | Age, gender, SES and maternal IQ |
|  |  | Unicoronal |  | -6.9 (-11.7, -2.1), *p*=0.006 | Age, gender, SES and maternal IQ |
|  |  | Lambdoid |  | -6.6 (-15.1, 1.9), *p*=0.13 | Age, gender, SES and maternal IQ |
|  | TOWRE | Metopic |  | -2.7 (-7.9, 2.4), *p*=0.30 | Age, gender, SES and maternal IQ |
|  |  | Unicoronal |  | -10.0 (-15.2, -4.7), p<0.001 | Age, gender, SES and maternal IQ |
|  |  | Lambdoid |  | -10.5 (-23, 2.0), p=0.10 | Age, gender, SES and maternal IQ |

† Only statistically significant results (*p*<0.05) are reported.

‡ Results with more than 50% difference in odds ratios are reported.

§ Short = upper secondary education, short or medium cycle higher education or bachelor’s degree.

¶ Long = master’s degree or Ph.D.

†† The associations between prevalence odds of special education use and type of OFC compared to control children for the 4^th^ and the 5^th^ grades were similar to those in the 3^rd^ grade and not reported in this table.

aOR, adjusted odds ratio; aPRR, adjusted prevalence rate ratio; CA, congenital anomaly; CHD, congenital heart defect; CI, confidence interval; CL, cleft lip; CLP, cleft lip and palate; CP, cleft palate; CPB, cardiopulmonary bypass; DHCA, deep hypothermic circulatory arrest; DMFT, decayed missing filled teeth; HC, hydrocephalus; IDACI, Income Deprivation Affecting Children Index; IQ, intelligence quotient; LBW, low birth weight; NA, not applicable; NAPLAN, National Assessment Program - Literacy and Numeracy; NHB, non-Hispanic Black; NHW, non-Hispanic White; NS, not significant (*p*≥0.05); OFC, orofacial cleft; OR, odds ratio; PR, prevalence ratio; RR, rate ratio; SB, spina bifida; SES, socioeconomic status; TOWRE, Test of Word Reading Efficiency; WALNA, Western Australian Literacy and Numeracy Assessment; WIAT, Wechsler Individual Achievement Test; WJTA-R, Woodcock-Johnson Tests of Achievement–Revised (percentiles); WJTA-R LWI, Woodcock-Johnson Tests of Achievement–Revised Letter-Word identification (age-based percentiles); WRAT, Wide Range Achievement Test.

**References**

Barf, H. A., Verhoef, M., Post, M. W., Jennekens-Schinkel, A., Gooskens, R. H., Mullaart, R. A., & Prevo, A. J. (2004). Educational career and predictors of type of education in young adults with spina bifida. *International Journal of Rehabilitation Research, 27*, 45-52. doi:10.1097/00004356-200403000-00006

Bell, J. C., Raynes-Greenow, C., Turner, R., Bower, C., Dodson, A., Nicholls, W., & Nassar, N. (2017). School performance for children with cleft lip and palate: a population-based study. *Child: Care, Health & Development, 43*, 222-231. doi:10.1111/cch.12388

Bellinger, D. C., Rivkin, M. J., DeMaso, D., Robertson, R. L., Stopp, C., Dunbar-Masterson, C., ... Newburger, J. W. (2015). Adolescents with tetralogy of Fallot: neuropsychological assessment and structural brain imaging. *Cardiology in the Young, 25*, 338-347. doi:10.1017/S1047951114000031

Broder, H. L., Richman, L. C., & Matheson, P. B. (1998). Learning disability, school achievement, and grade retention among children with cleft: a two-center study. *Cleft Palate-Craniofacial Journal, 35*, 127-131. doi:10.1597/1545-1569_1998_035_0127_ldsaag_2.3.co_2

Chapman, K. L. (2011). The relationship between early reading skills and speech and language performance in young children with cleft lip and palate. *Cleft Palate-Craniofacial Journal, 48*, 301-311. doi:10.1597/08-213

Clausen, N. G., Pedersen, D. A., Pedersen, J. K., Moller, S. E., Grosen, D., Wehby, G. L., ... Hansen, T. G. (2017). Oral Clefts and Academic Performance in Adolescence: The Impact of Anesthesia-Related Neurotoxicity, Timing of Surgery, and Type of Oral Clefts. *Cleft Palate-Craniofacial Journal, 54*, 371-380. doi:10.1597/15-185

Collett, B. R., Leroux, B., & Speltz, M. L. (2010). Language and early reading among children with orofacial clefts. *Cleft Palate-Craniofacial Journal, 47*, 284-292. doi:10.1597/08-172.1

Fitzsimons, K. J., Copley, L. P., Setakis, E., Charman, S. C., Deacon, S. A., Dearden, L., & van der Meulen, J. H. (2018). Early academic achievement in children with isolated clefts: a population-based study in England. *Archives of Disease in Childhood, 103*, 356-362. doi:10.1136/archdischild-2017-313777

Fitzsimons, K. J., Deacon, S. A., Copley, L. P., Park, M. H., Medina, J., & Van Der Meulen, J. H. (2021). School absence and achievement in children with isolated orofacial clefts. *Archives of Disease in Childhood, 106*, 154-159. doi:10.1136/archdischild-2020-319123

Fletcher, J. M., Copeland, K., Frederick, J. A., Blaser, S. E., Kramer, L. A., Northrup, H., ... Dennis, M. (2005). Spinal lesion level in spina bifida: a source of neural and cognitive heterogeneity. *Journal of Neurosurgery, 102*, 268-279. doi:10.3171/ped.2005.102.3.0268

Grewal, S. S., Ponduri, S., Leary, S. D., Wren, Y., Thompson, J. M. D., Ireland, A. J., ... Sandy, J. R. (2020). Educational Attainment of Children Born with Unilateral Cleft Lip and Palate in the United Kingdom. *Cleft Palate-Craniofacial Journal*, 1055665620959989. doi:10.1177/1055665620959989

Hiraiwa, A., Ibuki, K., Tanaka, T., Hirono, K., Miya, K., Yoshimura, N., & Ichida, F. (2020). Toddler neurodevelopmental outcomes are associated with school age IQ in children with single ventricle physiology. *Seminars in Thoracic and Cardiovascular Surgery, 32*, 302-310. doi:10.1053/j.semtcvs.2019.10.017

Lawley, C. M., Winlaw, D. S., Sholler, G. F., Martin, A., Badawi, N., Walker, K., ... Lain, S. J. (2019). School-Age Developmental and Educational Outcomes Following Cardiac Procedures in the First Year of Life: A Population-Based Record Linkage Study. *Pediatric Cardiology, 40*, 570-579. doi:10.1007/s00246-018-2029-y

Mahle, W. T., Clancy, R. R., Moss, E. M., Gerdes, M., Jobes, D. R., & Wernovsky, G. (2000). Neurodevelopmental outcome and lifestyle assessment in school-aged and adolescent children with hypoplastic left heart syndrome. *Pediatrics, 105*, 1082-1089. doi:10.1542/peds.105.5.1082

Mulkey, S. B., Bai, S., Luo, C., Cleavenger, J. E., Gibson, N., Holland, G., ... Bhutta, A. T. (2016). School-Age Test Proficiency and Special Education After Congenital Heart Disease Surgery in Infancy. *Journal of Pediatrics, 178*, 47-54. doi:10.1016/j.jpeds.2016.06.063

Olsen, M., Hjortdal, V. E., Mortensen, L. H., Christensen, T. D., Sorensen, H. T., & Pedersen, L. (2011). Educational achievement among long-term survivors of congenital heart defects: a Danish population-based follow-up study. *Cardiology in the Young, 21*, 197-203. doi:10.1017/S1047951110001769

Oster, M. E., Watkins, S., Hill, K. D., Knight, J. H., & Meyer, R. E. (2017). Academic Outcomes in Children With Congenital Heart Defects: A Population-Based Cohort Study. *Circulation: Cardiovascular Quality and Outcomes, 10*, e003074. doi:10.1161/CIRCOUTCOMES.116.003074

Riehle-Colarusso, T., Autry, A., Razzaghi, H., Boyle, C. A., Mahle, W. T., Van Naarden Braun, K., & Correa, A. (2015). Congenital Heart Defects and Receipt of Special Education Services. *Pediatrics, 136*, 496-504. doi:10.1542/peds.2015-0259

Saervold, T. K., Hide, O., Feragen, K. B., & Aukner, R. (2019). Associations Between Hypernasality, Intelligibility, and Language and Reading Skills in 10-Year-Old Children With a Palatal Cleft. *Cleft Palate-Craniofacial Journal, 56*, 1044-1051. doi:10.1177/1055665618824432

Schaefer, C. J., Hoop, R., Schurch-Reith, S., Stambach, D., Kretschmar, O., Bauersfeld, U., ... Landolt, M. A. (2016). Academic achievement and satisfaction in adolescents with CHD. *Cardiology in the Young, 26*, 257-262. doi:10.1017/S1047951115000074

Speltz, M. L., Collett, B. R., Wallace, E. R., Starr, J. R., Cradock, M. M., Buono, L., ... Kapp-Simon, K. (2015). Intellectual and academic functioning of school-age children with single-suture craniosynostosis. *Pediatrics, 135*, e615-623. doi:10.1542/peds.2014-1634

Watkins, S. E., Allori, A. C., Meyer, R. E., Aylsworth, A. S., Marcus, J. R., & Strauss, R. P. (2019). Special education use in elementary school by children with nonsyndromic orofacial clefts. *Birth Defects Research, 111*, 142-150. doi:10.1002/bdr2.1418

Watkins, S. E., Meyer, R. E., Aylsworth, A. S., Marcus, J. R., Allori, A. C., Pimenta, L. A., ... Strauss, R. P. (2018). Academic achievement among children with nonsyndromic orofacial clefts: A population-based study. *Cleft Palate-Craniofacial Journal, 55*, 12-20. doi:10.1177/1055665617718823

Wehby, G. L., Collet, B., Barron, S., Romitti, P. A., Ansley, T. N., & Speltz, M. (2014). Academic achievement of children and adolescents with oral clefts. *Pediatrics, 133*, 785-792. doi:10.1542/peds.2013-3072

Wills, K. E., Holmbeck, G. N., Dillon, K., & McLone, D. G. (1990). Intelligence and achievement in children with myelomeningocele. *Journal of Pediatric Psychology, 15*, 161-176. doi:10.1093/jpepsy/15.2.161

Yazdy, M. M., Autry, A. R., Honein, M. A., & Frias, J. L. (2008). Use of special education services by children with orofacial clefts. *Birth Defects Research, 82*, 147-154. doi:10.1002/bdra.20433
