# Supplementary material for: Academic achievement and needs of school‐aged children born with selected congenital anomalies: A systematic review and meta‐analysis
Source: Birth Defects Res. 2021 Oct 21;113(20):1431–62. doi: 10.1002/bdr2.1961 (PMC9298217; doi:10.1002/bdr2.1961)
Supplement: Supplementary file 7 — TABLE S7 Sensitivity analyses and tests for publication bias for papers included in the meta‐analysis of special education needs (SEN) in children with (1) congenital heart defects (CHD) and (2) orofacial clefts (OFC). [file BDR2-113-1431-s001.docx]

TABLE S7 Sensitivity analyses and tests for publication bias for papers included in the meta-analysis of special education needs (SEN) in children with 1) congenital heart defects (CHD) and 2) orofacial clefts (OFC)

1. **CHD – SEN**

**Sensitivity analysis**

|  | OR (95% CI) |  |
| --- | --- | --- |
| **Pooled** | **2.32 (1.90-2.82)** | **Significant increase** |
| Excluding Mulkey et al. (CHD with surgery at age <1 year) | 2.09 (1.82-2.41) | Weakest association |
| Excluding Oster et al. (critical CHD) | 2.39 (1.75-3.28) |  |
| Excluding Riehle-Colarusso et al. (critical CHD) | 2.52 (2.04-3.13) | Strongest association |
| Excluding Sarrechia et al. (univentricular heart disease) | 2.31 (1.90-2.81) |  |
| Excluding Wright et al. (cyanotic CHD with surgery at <2.5 years) | 2.30 (1.88-2.82) |  |

**Tests for publication bias**

**Egger’s test**

**H0: beta1 = 0; no small-study effects**

**beta1 = 0.67**

SE of beta1 = 0.809

z = 0.83

**Prob > |z| = 0.4043**

1. **OFC – SEN**

**Sensitivity analysis**

|  | OR (95% CI) |  |
| --- | --- | --- |
| **Pooled** | **2.74 (2.06-3.65)** | **Significant increase** |
| Excluding Watkins et al. (CL only) | 2.95 (2.26-3.84) | Strongest association |
| Excluding Yazdy et al. (CL only) | 2.84 (2.10-3.84) |  |
| Excluding Fitzsimons et al. (CL only) | 2.93 (2.22-3.88) |  |
| Excluding Collett et al. (CP only) | 2.74 (2.03-3.69) |  |
| Excluding Watkins et al. (CP only) | 2.69 (1.97-3.67) |  |
| Excluding Yazdy (CP only) | 2.69 (1.97-3.66) |  |
| Excluding Fitzsimons et al. (CP only) | 2.74 (1.99-3.76) |  |
| Excluding Collett et al. (CLP) | 2.66 (1.99-3.56) |  |
| Excluding Watkins et al. (CLP) | 2.61 (1.94-3.49) | Weakest association |
| Excluding Yazdy et al. (CLP) | 2.63 (1.95-3.55) |  |
| Excluding Fitzsimons et al. (CLP) | 2.68 (1.96-3.66) |  |
| Excluding Hentges et al. (CL and CLP) | 2.64 (1.98-3.53) |  |
| Excluding Wehby et al. (any OFC) | 2.89 (2.15-3.89) |  |

**Excluding subgroups**

|  | OR (95% CI) |  |
| --- | --- | --- |
| **Pooled** | **2.74 (2.06-3.65)** | **Significant increase** |
| Excluding subgroup 1 (CL only) | 3.34 (2.63-4.24) | Strongest association |
| Excluding subgroup 2 (CP only) | 2.60 (1.73-3.91) |  |
| Excluding subgroup 3 (CLP) | 2.25 (1.62-3.13) | Weakest association |
| Excluding subgroup 4 (Mixed OFC subtypes) | 2.78 (2.06-3.77) |  |

**Tests for publication bias**

**Egger’s test**

**H0: beta1 = 0; no small-study effects**

**beta1 = 0.93**

SE of beta1 = 1.001

z = 0.93

**Prob > |z| = 0.3517**
